# Integration of comprehensive genomic profiling, tumor mutational burden, and PD‐L1 expression to identify novel biomarkers of immunotherapy in non‐small cell lung cancer

**DOI:** 10.1002/cam4.3649

**Published:** 2021-03-02

**Authors:** Yunfei Shi, Youming Lei, Li Liu, Shiyue Zhang, Wenjing Wang, Juan Zhao, Songhui Zhao, Xiaowei Dong, Ming Yao, Kai Wang, Qing Zhou

**Affiliations:** ^1^ Department of geriatric thoracic surgery The First Hospital of Kunming Medical University Kunming City People's Republic of China; ^2^ Cancer Center Union Hospital Tongji Medical College Huazhong University of Science and Technology Wuhan People's Republic of China; ^3^ OrigiMed Shanghai People's Republic of China; ^4^ Guangdong Lung Cancer Institute Guangdong Provincial Key Laboratory of Translational Medicine in Lung Cancer Guangdong General Hospital and Guangdong Academy of Medical Sciences Guangzhou People's Republic of China

**Keywords:** lysine methyltransferase 2C, non‐small cell lung cancer, programmed cell death 1 ligand 1, tumor mutation burden, tumor protein p53

## Abstract

**Objectives:**

This study aimed to explore the novel biomarkers for immune checkpoint inhibitor (ICI) responses in non‐small cell lung cancer (NSCLC) by integrating genomic profiling, tumor mutational burden (TMB), and expression of programmed death receptor 1 ligand (PD‐L1).

**Materials and Methods:**

Tumor and blood samples from 637 Chinese patients with NSCLC were collected for targeted panel sequencing. Genomic alterations, including single nucleotide variations, insertions/deletions, copy number variations, and gene rearrangements, were assessed and TMB was computed. TMB‐high (TMB‐H) was defined as ≥10 mutations/Mb. PD‐L1 positivity was defined as ≥1% tumor cells with membranous staining. Genomic data and ICI outcomes of 240 patients with NSCLC were derived from cBioPortal.

**Results:**

*EGFR*‐sensitizing mutations, *ALK*, *RET*, and *ROS1* rearrangements were associated with lower TMB and PD‐L1+/TMB‐H proportions, whereas *KRAS*, *ALK*, *RET*, and *ROS1* substitutions/indels correlated with higher TMB and PD‐L1+/TMB‐H proportions than wild‐type genotypes. Histone‐lysine N‐methyltransferase 2 (*KMT2*) family members (*KMT2A*, *KMT2C*, and *KMT2D*) were frequently mutated in NSCLC tumors, and these mutations were associated with higher TMB and PD‐L1 expression, as well as higher PD‐L1+/TMB‐H proportions. Specifically, patients with KMT2C mutations had higher TMB and PD‐L1+/TMB‐H proportions than wild‐type patients. The median progression‐free survival (PFS) was 5.47 months (95% CI 2.5–NA) in patients with KMT2C mutations versus 3.17 months (95% CI 2.6–4.27) in wild‐type patients (*p* = 0.058). Furthermore, in patients with NSCLC who underwent ICI treatment, patients with TP53/KMT2C co‐mutations had significantly longer PFS and greater durable clinical benefit (HR: 0.48, 95% CI: 0.24–0.94, *p* = 0.033). TP53 mutation combined with KMT2C or KRAS mutation was a better biomarker with expanded population benefit from ICIs therapy and increased the predictive power (HR: 0.46, 95% CI: 0.26–0.81, *p* = 0.007).

**Conclusion:**

We found that tumors with different alterations in actionable target genes had variable expression of PD‐L1 and TMB in NSCLC. TP53/KMT2C co‐mutation might serve as a predictive biomarker for ICI responses in NSCLC.

**Implications for Practice:**

Cancer immunotherapies, especially immune checkpoint inhibitors (ICIs), have revolutionized the treatment of non‐small cell lung cancer (NSCLC); however, only a proportion of patients derive durable responses to this treatment. Biomarkers with greater accuracy are highly needed. In total, 637 Chinese patients with NSCLC were analyzed using next‐generation sequencing and IHC to characterize the unique features of genomic alterations and TMB and PD‐L1 expression. Our study demonstrated that KMT2C/TP53 co‐mutation might be an accurate, cost‐effective, and reliable biomarker to predict responses to PD‐1 blockade therapy in NSCLC patients and that adding KRAS to the biomarker combination creates a more robust parameter to identify the best responders to ICI therapy.

## Graphical abstract

Cancer immunotherapy, especially immune checkpoint inhibitors (ICIs), is epoch‐making in non‐small cell lung cancer (NSCLC), but only a proportion of patients can derive durable benefits from it, more accurate biomarkers are thus highly needed. Samples of 637 Chinese patients with NSCLC were analyzed through next‐generation sequencing and IHC to characterize the unique features of genomic alterations, TMB, and PD‐L1 expression. The results showed that the alteration subtypes of common driver genes including EGFR G719X, KRAS G12C, MET amplification, and SNVs/INDELs of ALK, RET, and ROS1 in NSCLC are associated with higher PD‐L1 expression or higher TMB value. Furthermore, our study first demonstrated that KMT2C/TP53 co‐mutation might be an accurate, cost‐effective, and reliable biomarker to predict the responses to PD‐1 blockade therapy in NSCLC patients, and that adding KRAS to the biomarker combination creates a more robust parameter to identify the best responders to ICI therapy.

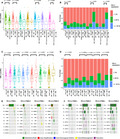

## 1 INTRODUCTION

Lung cancer is the most commonly diagnosed cancer and the leading cause of cancer‐related deaths in China.^1^ Immune checkpoint inhibitors (ICIs), particularly inhibitors of the programmed cell death 1 (PD‐1)/PD‐1 ligand (PD‐L1) axis, have altered the landscape of non‐small cell lung cancer (NSCLC) treatment.^2^ Responses to ICIs can be remarkably durable, but they only occur in a minority of patients,^3^ causing the determinants of ICI sensitivity to remain elusive. Therefore, it has become an urgent priority to identify the biomarkers of responsiveness to ICIs and develop strategies that could potentially increase the patient response rates or the number of patients who may potentially benefit from ICI treatment.

Several ICI‐responsiveness biomarkers have been proposed, including the tumoral expression of PD‐L1,^4^ tumor‐infiltrating lymphocytes (TILs),^5^ tumor mutational burden (TMB),^6^, ^7^ DNA mismatch‐repair deficiency,^8^ and gene signatures reflecting preexisting adaptive immunity ^9^; however, each of these biomarkers has limited utility. Although PD‐L1 immunohistochemistry (IHC) is the first Food and Drug Administration‐approved companion diagnostic test for ICI treatment, challenges in defining the positive thresholds during quantification, the lack of consensus regarding the anti‐PD‐L1 antibodies used, and intratumoral heterogeneity limit the clinical application of PD‐L1 detection.^10^ TMB is another potential predictive factor of the ICI response. An association between an elevated TMB and the response to or durable clinical benefits (DCBs) of ICIs in patients with NSCLC was identified in several clinical trials^11^ and real‐world studies.^6^, ^7^ However, the use of TMB as a biomarker requires expensive genomic platforms and long turnaround times; moreover, TMB has no validated cutoff value, which limits the use of this biomarker.^12^ Importantly, the main limitation of strategies employing biomarkers is linked to the selection of an optimal cutoff for clinical decision making.

Somatic mutations in specific genes may influence the ability of tumor cells to evade immune surveillance. Sensitizing mutations in the epidermal growth factor receptor (*EGFR*) gene and rearrangements in the anaplastic lymphoma kinase (*ALK*) gene are predictors of a poor response to ICIs, and patients with these genomic alterations are routinely excluded from most ICI trials.^13^ A retrospective analysis has found that only 3.6% of patients with such gene alterations responded to ICIs, while 23.3% of *EGFR* wild‐type and *ALK*‐negative patients and patients with an unknown genetic background responded to ICIs.^14^ A meta‐analysis of five clinical trials that included 3,025 patients with advanced NSCLC who were treated with a PD‐L1 inhibitor found that, among patients with *EGFR* mutations, the overall survival (OS) was not improved compared to those treated with docetaxel.^15^ *EGFR*‐mutated or *ALK*‐rearranged lung cancers exhibit lower PD‐L1 expression levels and lower CD8^+^ T‐cell infiltration, which may be responsible for the poor response to ICIs.^16^ In contrast, genomic alterations in DNA damage response pathways, mainly including homologous recombination repair, mismatch repair (MMR), and DNA polymerase ε (*POLE*)/DNA polymerase δ (*POLD*) genes,^17^ are associated with an increase in TMB and have been identified as predictors of favorable responses to ICIs.^18^ A subset of patients with KRAS proto‐oncogene (*KRAS*)/tumor protein p53 (*TP53*) mutant NSCLC were shown to be characterized by increased expression of PD‐L1 in tumor cells, increased TMB, and a higher degree of tumor infiltration by CD8^+^ T cells, which led to improved clinical outcomes.^19^ In contrast, co‐mutations in serine/threonine kinase 11 (*STK11*) in *KRAS*‐mutated lung tumors were associated with low expression of PD‐L1 and a decreased response to ICIs, leading to a poor survival.^20^ More recently, a subgroup of patients with NSCLC was found to carry *TP53* and ataxia telangiectasia‐mutated (*ATM*) co‐mutations, which were associated with an increased TMB and a better response to ICIs.^21^ These findings raise questions regarding whether comprehensive genomic alterations in tumor tissues are related to existing ICI biomarkers, such as PD‐L1 and TMB, and whether other specific genes can predict the therapeutic outcomes.

Epigenetic dysregulation, including DNA methylation, histone modifications, and noncoding RNAs, has been reported to be involved in the pathogenesis of NSCLC and responses to immunotherapy.^22^ A prospective, multicenter study has associated a specific epigenetic profile, based on DNA methylation microarrays, with progression‐free survival (PFS) and OS in patients with NSCLC, who were receiving anti‐PD‐1 antibodies.^23^ Histone methylation is a particularly important process that regulates the transcription of genes associated with the evasion of immune surveillance by tumors. Histone‐lysine N‐methyltransferase 2 (KMT2) family proteins methylate lysine 4 on the histone H3 tail in important genomic regulatory regions and thereby modulate the chromatin structure and DNA accessibility.^24^ Mutations in genes of the *KMT2* family are among the most frequent in human cancer and are associated with some of the most common and deadly solid tumors, such as lung^25^ and colon^26^ carcinomas. The KMT2C protein, also known as MLL3, is part of a transcriptional coactivator complex and a tumor suppressor involved in several cellular processes, including the regulation of homeostasis and hormone receptor signaling.^27^ Notably, *KMT2C* is frequently mutated in cancers, including lung adenocarcinoma,^28^ but the association between KMT2C and immunotherapeutic responses remains unclear. Therefore, we hypothesized that the mutational status of *KMT2C* or *KMT2C*‐based mutational signatures might be useful as a predictive biomarker in ICI‐treated patients with NSCLC.

In this study, a cohort comprising 637 patients with NSCLC was used to determine the landscape of immune biomarkers in patients with genomic alterations, including those in NSCLC driver genes and other frequently mutated genes, as detected by next‐generation sequencing (NGS). Furthermore, we validated our hypothesis using data of clinical immunotherapeutic patients from the cBioPortal database. We demonstrated that *TP53*/*KMT2C* co‐mutations predict a better response to ICI therapies and that the *TP53* mutation, in conjunction with either a *KRAS* or *KMT2C* mutation, could be used as a potential predictor of the ICI response. These biomarkers could allow a greater number of patients to benefit from ICI treatment.

## 2 MATERIALS AND METHODS

### 2.1 Patient selection

In total, 637 patients who received surgery or were biopsied from December 2017 to January 2019, with a final pathological diagnosis of NSCLC, were retrospectively identified in this study. Tumor samples were collected between December 2017 and January 2019. Matched blood samples were collected as normal controls. Tissue samples were formalin‐fixed paraffin‐embedded (FFPE) in accredited clinical hospitals with 10% formalin for 24–72 hours at room temperature. This study was approved by the Institution Review Board of the First Hospital of Kunming Medical University and Guangdong Provincial People's Hospital and conducted according to the Declaration of Helsinki. Informed consent was obtained from all enrolled patients.

To further explore the association between gene mutations and the clinical benefit of ICIs, we included genomic and clinical data derived from the cohort treated with immune checkpoint blockades (ICBs) in cBioPortal (www.cbioportal.org),^29^, ^30^ which consisted of 240 patients with NSCLC treated with an anti‐PD‐1 therapeutic scheme (Rizvi cohort).^31^ Measures of PFS and durable clinical benefit (complete response/partial response or stable disease that lasted >6 months) were based on definitions consistent with those in the abovementioned trial.

### 2.2 NGS and bioinformatics analysis

All tumor tissues and matched blood samples underwent targeted NGS‐based genomic testing (OrigiMed, Shanghai, China) in a College of American Pathologists (CAP)‐accredited and Clinical Laboratory Improvement Amendments (CLIA)‐certified laboratory.^32^ Approximately 50 ng of cancer tissue DNA was extracted from 40 mm FFPE and blood samples using the DNA Extraction Kit (QIAamp DNA FFPE Tissue Kit, Cat no. 60404; Qiagen), according to the manufacturer's instructions. All coding exons and selected introns of targeted genes were captured using a hybridization capture panel and then, sequenced on an Illumina NextSeq‐500 Platform (Illumina Incorporated). For FFPE samples the sequencing depth mean coverage was 900× (minimum 700×), while for matched blood samples the sequencing depth was 300×. Genomic alterations, including single nucleotide variants (SNVs), short and long insertions/deletions (indels), copy number variations (CNVs), and gene rearrangements were subjected to advanced analysis. TMB was defined as somatic mutations including coding base substitutions and indel mutations per megabase (muts/Mb) of genome examined. For our TMB calculation, all indels and non‐synonymous alterations in the coding region were considered while known hotspot mutations in oncogenic drivers and known germline alterations in the single nucleotide polymorphism database (dbSNP) were excluded.^33^ High TMB (TMB‐H) and low TMB (TMB‐L) were defined as ≥10 and <10 muts/Mb, respectively.^34^ The functionality of *KMT2C* mutations was assessed by in silico mutation prediction tools, namely PolyPhen‐2 (http://genetics.bwh.harvard.edu/pph2/). Mutational signature analysis was carried out based on R packages “deconstructSigs” (version 1.8.0) and “SomaticSignatures” (version 3.11) using 30 COSMIC signatures.^35^, ^36^

### 2.3 PD‐L1 IHC assessment

PD‐L1 IHC staining assay was performed as previously described.^37^ The expression of PD‐L1 was assessed by IHC analysis of FFPE tumor samples using anti‐PD‐L1 antibodies (clone 22C3; Cat no. M3653; Dako). The PD‐L1 tumor proportion score (TPS), which is the percentage of tumor cells showing partial or complete membrane staining, was determined and classified as negative, low‐positive, or high‐positive (TPS of <1%, 1%–49%, and ≥50%, respectively).

### 2.4 Statistical analysis

Statistical analysis was conducted using the R Statistical Software package (version 3.4.3, R Foundation for Statistical Computing). Categorical variables are presented as numbers and percentages; medians and percentiles are reported for continuous variables. In multiple‐group comparisons, Kruskal–Wallis rank sum tests, Chi‐square tests, or Fisher's exact tests (limited sample size [<10] for single category), with Bonferroni post hoc comparisons were used. Kendall's rank correlations were used to correlate the expression levels of PD‐L1 and TMBs. Survival analysis was performed using Kaplan–Meier curves, with *p* values determined by log‐rank tests. Hazard ratios (HRs) were determined through Cox regression. The threshold for statistical significance was set at *p* < 0.05.

## 3 RESULTS

### 3.1 Correlation between expression of PD‐L1, TMB, and clinicopathologic characteristics

In total, 637 patients with NSCLC were included in the study. Clinical characteristics of patients are shown in Table S1. The majority of patients were male (355/637; 55.7%) and the median age at diagnosis was 60 years (interquartile range [IQR], 53–67 years). Non‐squamous cell carcinoma was the most frequent histological type (N = 553, 86.8%), including adenocarcinoma (N = 537) and other non‐squamous histological subtypes (N = 16).

Expression of PD‐L1 and TMB was assessed in all 637 patients. The distribution of the expression of PD‐L1 and TMB according to demographic characteristics is presented in Table S1. Representative IHC stains of PD‐L1 in NSCLC using 22C3 are shown in Figure S1. Low‐positive (TPS =1–49%) and high‐positive (TPS ≥50%) expression of PD‐L1 was observed in 16.5% and 10% of cases, respectively. We performed univariate analysis of the association of expression of PD‐L1 (evaluated as categorical variables with cut‐off values of 1% and 50%) with clinical features of NSCLC (Table S1). Expression of PD‐L1 was significantly higher in males (*p* = 0.002) and in squamous cell carcinomas (*p* < 0.001).

The median TMB for the cohort was 4.6 muts/Mb (IQR, 2.3–10). A TMB ≥10 muts/Mb was seen in 29.4% of tumors. We found a significant increase in age‐associated with higher TMB (*p* < 0.001). TMB was significantly higher in males (*p* < 0.001), squamous cell carcinomas (*p* < 0.001), and current/former smokers. Finally, there was a weak association between the expression of PD‐L1 and TMB (Kendall's coefficient 0.179, *p* < 0.001). (Figure S2); the median IQR of TMB was 4.25 (2.3–8.5), 6.9 (3.8–13.1), and 9.2 (3.6–13.9) for PD‐L1 TPS <1%, 1–49%, and ≥50%, respectively (*p* < 0.001).

### 3.2 Correlation between expression of PD‐L1, TMB, and gene alterations

Overall, the average number of mutations per cancer sample was 7.9. The incidence of genomic alterations and the mutational profile (frequency and composition) are summarized in Figure S3. The frequency and composition of genomic alterations in lung cancer‐related genes were different between squamous and non‐squamous cell carcinomas. Considering the histological subtypes of NSCLC, non‐squamous and squamous lung cancers displayed different patterns. The frequency of the driver gene *EGFR* alterations was much higher in non‐squamous lung cancers than in squamous lung cancers (57.5% vs. 10.7%, *p* < 0.001). In contrast, the frequency of *KMT2C* and *CDKN2A* was lower in non‐squamous lung cancers (*KMT2C*: 4.2% vs. 10.7%, *p* = 0.021; *CDKN2A*: 10.1% vs. 25%, *p* < 0.001). Similarly, the frequency of phosphatidylinositol‐4, 5‐bisphosphate 3‐kinase catalytic subunit alpha (*PIK3CA*) changes was much lower in non‐squamous lung cancers (8% vs. 40.5%, *p* < 0.001). However, the prevalence of catenin beta 1 (*CTNNB1*) and cyclin‐dependent kinase 4 (*CDK4*) only showed alterations in non‐squamous, but not in squamous lung cancers. Of interest, *EGFR* mutations (51.3%) and *ALK* fusion/rearrangements (5.8%) were the most frequent actionable genomic alterations in our NSCLC cohort. Additional actionable alterations were: erb‐b2 receptor tyrosine kinase 2 (*ERBB2*) mutations (4.1%), B‐Raf proto‐oncogene (*BRAF*) V600E (1.3%), MET proto‐oncogene (*MET*) amplification (1.6%), *MET* ex14 skipping (0.8%), Ret proto‐oncogene (*RET*) fusion/rearrangements (1.4%), and ROS proto‐oncogene 1 (*ROS1*) fusion/rearrangements (2%). Other frequent genomic alterations were those involving *TP53* (55.9%), *KRAS* (12.6%), *PIK3CA* (12.2%), LDL receptor‐related protein 1B (*LRP1B*) (12.1%), *CDKN2A* (12.1%), RNA‐binding motif protein 10 (*RBM10*) (12.1%), and telomerase reverse transcriptase (*TERT*) (11.3%). To gain unique insights into the mutational processes in Chinese patients with NSCLC, we performed the mutational signature analysis of the somatic mutation data from the OrigiMed cohort, and the overall mutational pattern is shown in Figure S4A. Our analysis identified that aging, BRCA1/2 deficiency, MMR, and smoking signatures were four major mutational signatures in the OrigiMed cohort (Figure S4B).

**FIGURE 1:**
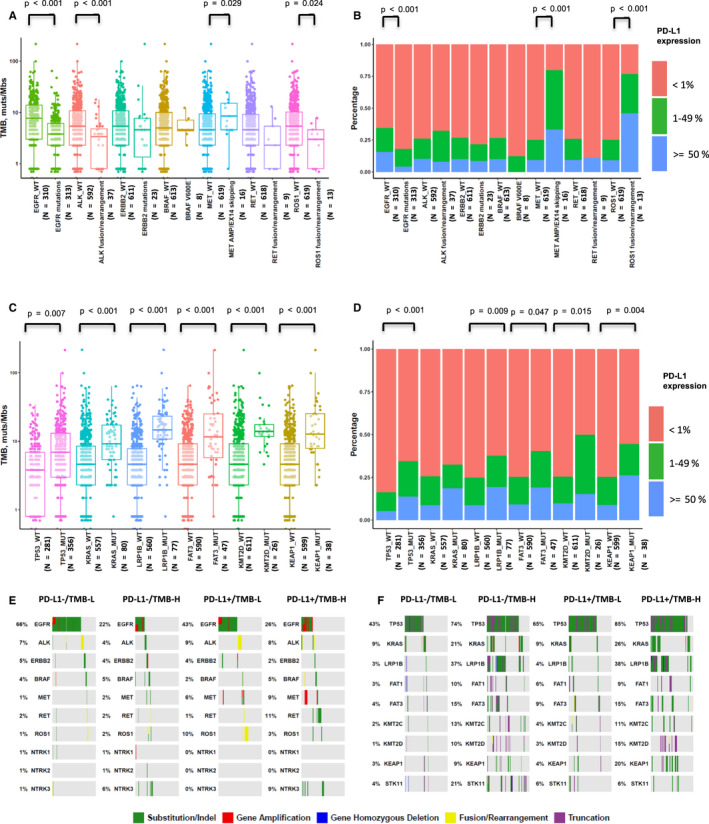
Correlations between expression of PD‐L1, TMB, and gene alterations. (A) Comparison of TMB between actionable‐mutated and wild‐type genes, including *EGFR*, *ALK*, *ERBB2*, *BRAF*, *MET*, *RET*, and *ROS1*. A box‐and‐whisker plot is used to represent the data. Box plot represents first (lower bound) quartile, median, and third (upper bound) quartile. Whiskers, representing 1.5 times the interquartile range, were used to visualize data for these comparisons. Kruskal–Wallis rank sum tests were used for comparisons of TMB between two groups with or without corresponding genomic alterations. Dots represent individual tumors. (B) Comparison of expression of PD‐L1 between actionable‐mutated and wild‐type genes, including *EGFR*, *ALK*, *ERBB2*, *BRAF*, *MET*, *RET*, and *ROS1*. Chi‐square tests were used for comparisons of PD‐L1 expression between two groups with or without *EGFR* genomic alterations. Fisher's exact tests were used for comparisons of PD‐L1 expression between two groups with or without *ALK*, *ERBB2*, *BRAF*, *MET*, *RET*, and *ROS1* genomic alterations. (C) Comparison of TMB between mutated and wild‐type genes, including *TP53*, *KRAS*, *LRP1B*, *FAT3*, *KMT2D*, and *KEAP1*. (D) Comparison of expression of PD‐L1 between mutated and wild‐type genes, including *TP53*, *KRAS*, *LRP1B*, *FAT3*, *KMT2D*, and *KEAP1*. Chi‐square tests were used for comparisons of PD‐L1 expression between two groups with or without corresponding genomic alterations. (E, F) OncoPrint depicting alterations in preselected genes of interest in groups defined by a composite variable of TMB (stratified above and below 10 muts/Mb) and expression of PD‐L1 (stratified above and below 1% TPS).

Genomic alterations that were associated with TMB and expression of PD‐L1 at the univariate level are depicted in Figure 1. Among common actionable mutations, *MET* actionable alterations (*MET* amplification and exon 14 skipping) were significantly, positively correlated with TMB (*p* = 0.029), whereas *EGFR* actionable mutations (L858R, exon 19 deletion, exon 20 insertion, G719X, L861Q, and T790 M), *ALK* rearrangements, and *ROS1* rearrangements were significantly, negatively correlated with TMB (*p* < 0.001, *p* = 0.000018, and *p* = 0.024, respectively) (Figure 1A). Concomitantly, *MET* amplification/ex14 skipping and *ROS1* rearrangements were significantly, positively correlated with the expression of PD‐L1 (*p* < 0.001 and *p* < 0.001, respectively), whereas *EGFR* actionable mutations were significantly, negatively correlated with the expression of PD‐L1 (*p* < 0.001) (Figure 1B).

In addition, *TP53*, *KRAS*, *LRP1B*, FAT atypical cadherin 3 (*FAT3*), *KMT2D*, and kelch like ECH‐associated protein 1 (*KEAP1*) mutations occurred frequently in patients with NSCLC (Table S3) and were significantly, positively correlated with TMB (*p* = 0.007, *p* < 0.001, *p* < 0.001, *p* < 0.001, and *p* < 0.001, respectively), and with the expression of PD‐L1 (*p* < 0.001, *p* = 0.009, *p* = 0.047, *p* = 0.015, and *p* = 0.004, respectively) (Figure 1C and 1D).

To further determine whether molecular alterations were associated with the expression of PD‐L1 and TMB, we classified our patients into four categories, based on expression of PD‐L1 (≥1%) and TMB value (≥10 muts/Mb). We investigated the distribution of gene alterations in each category, including that of common actionable mutations and several frequent mutations in NSCLC (Figure 1E and 1F, Tables S2 and S3). Noted, *TP53* mutations were enriched in the PD‐L1+/TMB‐H group (Figure 1F), whereas *EGFR* mutations were enriched in the PD‐L1−/TMB‐L group (Figure 1E). Moreover, *ROS1* rearrangements were enriched in the PD‐L1+/TMB‐L group (Figure 1E), whereas *STK11* mutations were enriched in the PD‐L1−/TMB‐H group (Figure 1F).

### 3.3 Correlation between expression oF PD‐L1, TMB, and mutational status of *KMT2* family genes

**FIGURE 2:**
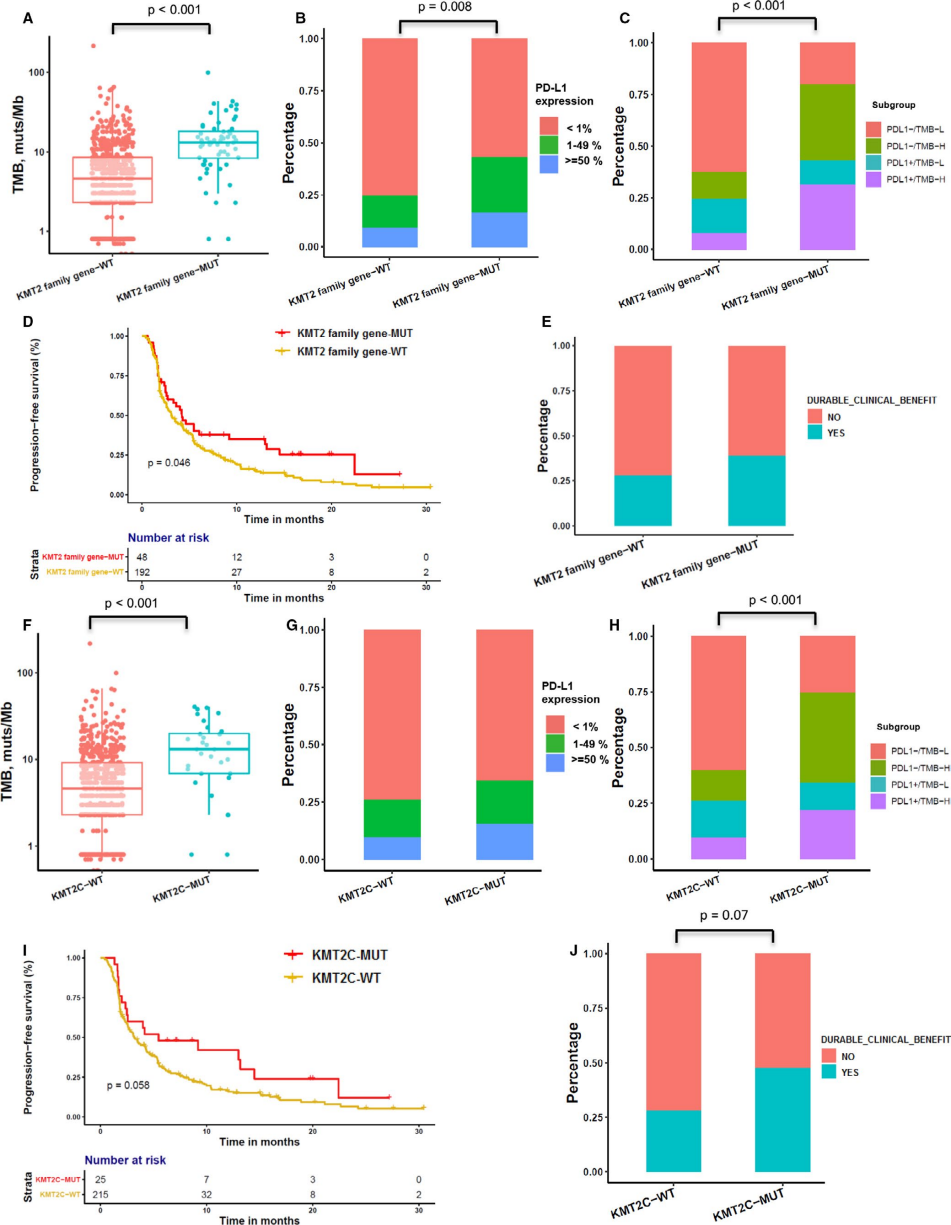
Correlation between KMT2 family gene mutations and PD‐L1, TMB, and clinical response to ICIs. (A) Comparison of TMB among groups classified by KMT2 family gene (*KMT2A*, *KMT2C*, and *KMT2D*) mutational status. A box‐and‐whisker plot is used to represent the data. Box plot represents first (lower bound) quartile, median, and third (upper bound) quartile. Whiskers, representing 1.5 times the interquartile range, were used to visualize data for these comparisons. Kruskal–Wallis rank sum tests were used for comparisons of TMB between two groups with or without KMT2 family gene genomic alterations. Dots represent individual tumors. (B) Comparison of expression of PD‐L1 among groups classified by KMT2 family gene (*KMT2A*, *KMT2C*, and *KMT2D*) mutational status. Chi‐square tests were used for comparisons of PD‐L1 expression between two groups with or without KMT2 family gene genomic alterations. (C) Comparison of distribution of PD‐L1−/TMB‐L, PD‐L1−/TMB‐H, PD‐L1+/TMB‐L, and PD‐L1+/TMB‐H groups among groups classified by KMT2 family gene (*KMT2A*, *KMT2C*, and *KMT2D*) mutational status. Fisher's exact tests were used for comparisons of distribution of four subgroups between two groups with or without KMT2 family gene genomic alterations. (D) Kaplan–Meier survival curves of PFS comparing patients with KMT2 family gene mutations with wild‐type patients, both treated with ICIs. Log‐rank tests were used for comparisons of PFS between two groups with or without KMT2 family gene genomic alterations. (E) Histogram depicting the DCB proportions among patients in groups defined by KMT2 family gene mutation status. Chi‐square tests were used for comparisons of DCB rate between two groups with or without KMT2 family gene genomic alterations. (F) Comparison of TMB among groups classified by *KMT2C* gene mutational status. (G) Comparison of expression of PD‐L1 among groups classified by *KMT2C* gene mutational status. Fisher's exact tests were used for comparisons of PD‐L1 expression between two groups with or without *KMT2C* genomic alterations. (H) Comparison of distribution of PD‐L1−/TMB‐L, PD‐L1−/TMB‐H, PD‐L1+/TMB‐L, and PD‐L1+/TMB‐H groups among those classified by *KMT2C* gene mutational status. Fisher's exact tests were used for comparisons of distribution of four subgroups between two groups with or without *KMT2C* genomic alterations. (I) Kaplan–Meier survival curves of PFS comparing patients with *KMT2C* gene mutations with wild‐type patients, both treated with ICIs. (J) Histogram depicting the DCB proportions among patients in groups defined by *KMT2C* gene mutation status. Chi‐square tests were used for comparisons of DCB rate between two groups with or without *KMT2C* genomic alterations.

We also evaluated whether mutations in individual altered genes were associated with TMB (stratified as ≥10 muts/Mb vs. <10 muts/Mb) and expression of PD‐L1 (stratified as ≥1% vs. <1%). As shown in Figure S5, *LRP1B* and *TP53* were the most enriched genes in the TMB‐H or PD‐L1+ cohort. Furthermore, *KMT2* family genes, such as *KMT2A*, *KMT2C*, and *KMT2D*, were frequently mutated in TMB‐H or PD‐L1+ tumor samples (Figure S5A and S5B). The mutational profile (frequency and composition) of *KMT2* family genes (*KMT2A* [1.3%], *KMT2C* [5%], and *KMT2D* [4%]) is shown in Figure S5C. Tumors with *KMT2* family gene mutations (*KMT2A*, *KMT2C*, and *KMT2D*) had significantly higher TMB (13.1 vs. 4.6 muts/Mb, *p* < 0.001), PD‐L1+ proportions (43.3% vs. 24.8%, *p* = 0.008), and PD‐L1+/TMB‐H proportions (37.7% vs. 8%, *p* < 0.001) than those without *KMT2* family gene mutations (Figure 2A–C). We further tested the association of *KMT2* family gene mutations with the clinical outcomes of ICI treatment in the Rizvi cohort.^31^ This cohort included 240 patients with NSCLC treated with anti‐PD‐L1 alone or in combination with anti‐cytotoxic T‐cell lymphocyte‐4 (anti‐CTLA‐4) therapeutic scheme. The PFS of 48 patients classified as *KMT2* family gene‐MUT was superior to that of the wild‐type *KMT2* family gene patients (median, 4.2 [95% CI: 2.57–13] vs. 3.1 [95% CI: 2.57–4.33] months, *p* = 0.046) (Figure 2D). However, there was no significant difference in the rate of DCB in patients with or without *KMT2* family gene mutations (39% vs. 28%, *p* = 0.2) (Figure 2E).

Tumors with *KMT2C* mutations had significantly higher TMB and PD‐L1+/TMB‐L proportions compare to *KMT2C* wild‐type tumors (Figure 2F and 2H), but there was no significant difference in the expression of PD‐L1 between the two groups (Figure 2G). In the Rizvi cohort, the PFS of 25 patients classified as *KMT2C*‐MUT was superior to that of the *KMT2C*‐WT patients (median, 5.47 vs. 3.17 months, *p* = 0.058) (Figure 2I). Due to the small patient number in the *KMT2C*‐MUT subgroup, the study only showed a trend toward a higher rate of DCB relative to the *KMT2C*‐WT subgroup (48% vs. 28%, respectively; *p* = 0.07) (Figure 2J). Although *KMT2A* and *KMT2D* mutations correlated with higher TMB and PD‐L1+/TMB‐L proportions (Figure S6A‐F), patients with these mutations did not exhibit improved PFS over wild‐type *KMT2A* or *KMT2D* patients (Figure S6G‐H). *KMT2C* mutations found in patients with NSCLC and in silico mutation prediction analyses performed for the OrigiMed and cBioportal data sets using PolyPhen‐2 are summarized in Tables S4 and S5.

### 3.4 Relevance of *KMT2C*/*TP53* co‐mutation status to expression of PD‐L1 and TMB as a predictive biomarker

**FIGURE 3:**
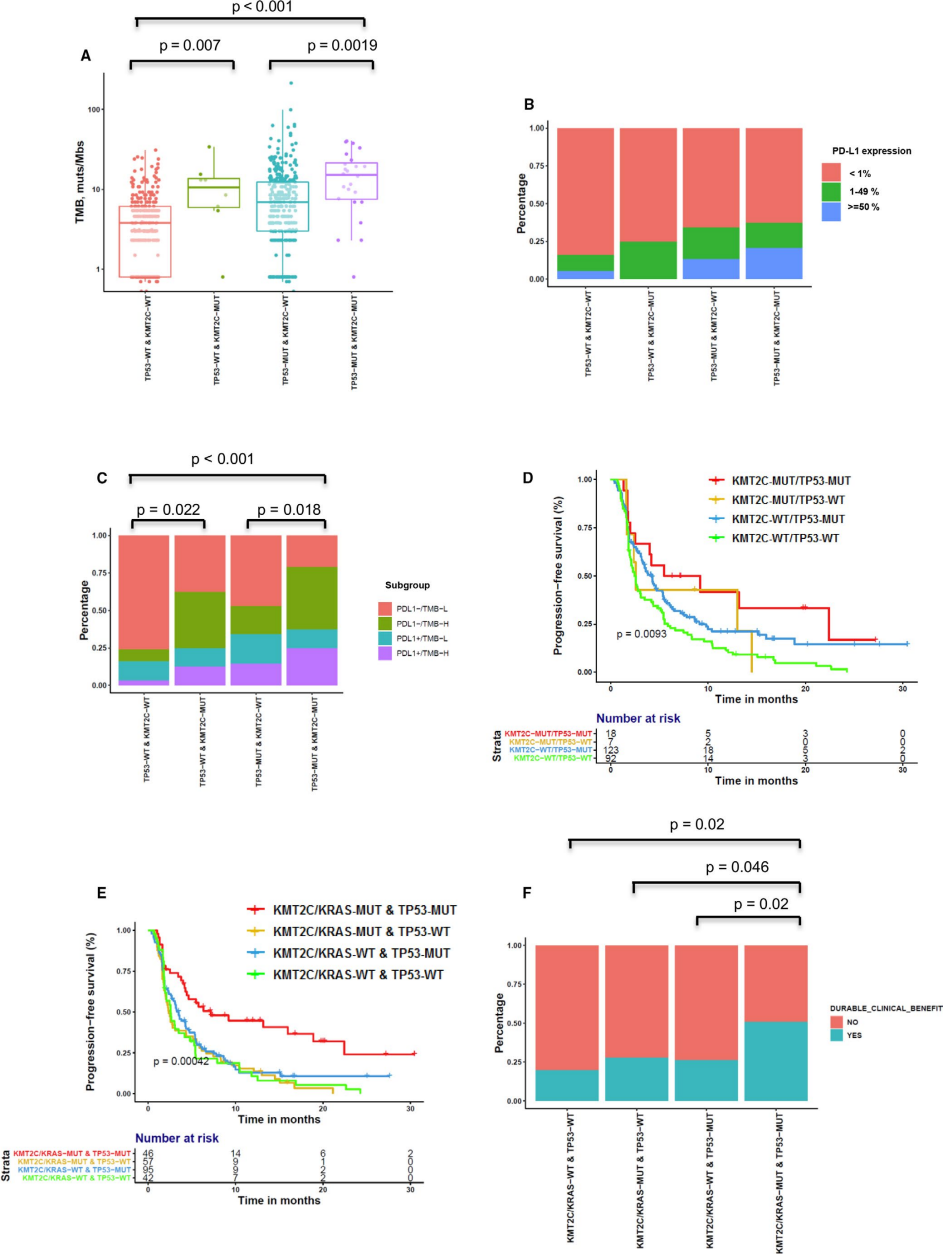
Correlation between *KMT2C*/*TP53* mutation and PD‐L1, TMB, and clinical response to ICIs. (A) Comparison of TMB among groups classified by *KMT2C* and *TP53* mutational status. A box‐and‐whisker plot is used to represent the data. Box plot represents first (lower bound) quartile, median, and third (upper bound) quartile. Whiskers, representing 1.5 times the interquartile range, were used to visualize data for these comparisons. Kruskal–Wallis rank sum tests were used for comparisons of TMB across four groups. Dots represent individual tumors. (B) Comparison of expression of PD‐L1 among groups classified by *KMT2C* and *TP53* mutational status. Fisher's exact tests were used for comparisons of PD‐L1 expression between four groups classified by *KMT2C* and *TP53* mutational status. (C) Comparison of distribution of PD‐L1−/TMB‐L, PD‐L1−/TMB‐H, PD‐L1+/TMB‐L, and PD‐L1+/TMB‐H groups among those classified by *KMT2C* and *TP53* mutational status. Fisher's exact tests were used for comparisons of PD‐L1 expression across four groups. (D) Kaplan–Meier survival curves of PFS comparing patients with *TP53* or *KMT2C* mutations with wild‐type patients, both treated with ICIs. Log‐rank tests were used for comparisons of PFS across four groups. (E) Kaplan–Meier survival curves of PFS comparing patients with *TP53*/*KMT2C*/*KRAS* mutations with wild‐type patients, both treated with ICIs. (F) Histogram depicting the DCB proportions among patients in groups defined by *KMT2C*, *KRAS*, and *TP53* mutation status. Fisher's exact tests were used for comparisons of DCB rate between four groups classified by *KMT2C* and *TP53* mutational status.

As *TP53* was frequently mutated in patients with *KMT2C* mutations in the OrigiMed (75%) and Rizvi (72%) cohorts, we further confirmed the synergetic effect of epigenetic‐related genes and tumor suppressor gene mutations as predictive biomarkers for cancer immunotherapies. Tumors with *KMT2C* mutations had significantly higher TMB and PD‐L1+/TMB‐H proportions than *KMT2C* wild‐type tumors, irrespective of *TP53* aberrances (Figure 3A and 3C), but there was no significant difference in the expression levels of PD‐L1 between the four subgroups (Figure 3B). We further tested the association of *KMT2C*/*TP53* co‐mutation status with the clinical outcomes of ICI therapy in the Rizvi cohort.^31^

Briefly, 18, 7, 123, and 92 patients were classified as *KMT2C*/*TP53* co‐MUT, *TP53*‐WT & *KMT2C*‐MUT, *TP53*‐MUT & *KMT2C*‐WT, and *KMT2C*/*TP53* co‐WT subgroups, respectively. Additionally, the corresponding median PFS (95% CI) was 7.33 months (2.5–NA), 2.57 months (1.63–NA), 4.2 months (3.23–5.37), and 2.47 months (2.03–3.5) (*KMT2C*/*TP53* co‐MUT vs. *KMT2C*/*TP53* co‐WT, adjusted HR: 0.48, 95% CI: 0.24–0.94, *p* = 0.0327; *TP53*‐MUT & *KMT2C*‐WT vs. *KMT2C*/*TP53* co‐WT, HR: 0.72, 95% CI: 0.53–0.99, *p* = 0.049), respectively (Figure 3D and Table 1). Moreover, the *KMT2C*/*TP53* co‐MUT subgroup tended to show a higher rate of DCB relative to the *TP53*‐WT & *KMT2C*‐MUT, *TP53*‐MUT & *KMT2C*‐WT, and *KMT2C*/*TP53* co‐WT subgroups (50%, 42.8%, 32.1%, and 23.3%, respectively; *p* = 0.06) (Figure S7A).

**TABLE 1:** Univariate and multivariate analyses of clinical parameters and co‐mutation status of *KMT2C* and *TP53* on progression‐free survival.

	Unadjusted HR (95% CI)	*p* value	Adjusted HR (95% CI)	*p* value
Gender				
Female	Reference		Reference	
Male	1.09 (0.83–1.44)	0.5420	1.13 (0.85–1.51)	0.4051
Age	1.00 (0.99–1.01)	0.9270	0.99 (0.98–1.01)	0.4144
Smoking status				
Ever	Reference		Reference	
Never	1.45 (1.03–2.03)	0.0321	1.41 (0.96–2.06)	0.0795
Treatment type				
Combination	Reference		Reference	
Monotherapy	1.87 (1.21–2.87)	0.0045	2.03 (1.30–3.18)	0.0018
TMB group				
<10 muts/Mb	Reference		Reference	
>=10 muts/Mb	0.73 (0.54–0.99)	0.0454	0.98 (0.69–1.40)	0.9142
Histological type				
Non‐Squamous	Reference		Reference	
Squamous	1.02 (0.68–1.53)	0.9240	0.96 (0.63–1.45)	0.8319
Co‐mutation				
KMT2C‐WT/TP53‐WT	Reference		Reference	
KMT2C‐MUT/TP53‐WT	0.72 (0.31–1.65)	0.4389	0.83 (0.36–1.92)	0.6595
KMT2C‐WT/TP53‐MUT	0.68 (0.51–0.91)	0.0092	0.72 (0.53–0.99)	0.0490
KMT2C‐MUT/TP53‐MUT	0.44 (0.24–0.80)	0.0076	0.48 (0.24–0.94)	0.0327

As we have used PolyPhen‐2 to predict the functional impact of the *KMT2C* mutation in the OrigiMed and cBioportal data sets (Table S4–S5), We further reassessed the genomic and survival analysis with only potentially damaging *KMT2C* mutations, the results showed that predicted loss‐of‐function *KMT2C* mutations were associated with higher TMB but not correlated with PD‐L1 expression (Figure S8A‐S8B) and patients with potentially damaging *KMT2C* mutations had a trend for better PFS than those *KMT2C* wild‐type patients (median, 4.08 vs. 3.17 months, *p* = 0.25), which was consistent with data in Figure 2 (Figure S8C). We further reassessed the association of *KMT2C* (potentially damaging mutations)/*TP53* co‐mutation status with the clinical outcomes of ICI therapy in the Rizvi cohort. Briefly, 15, 5, 123, and 92 patients were classified as *KMT2C*/*TP53* co‐MUT, *TP53*‐WT & *KMT2C*‐MUT, *TP53*‐MUT & *KMT2C*‐WT, and *KMT2C*/*TP53* co‐WT subgroups, respectively. Additionally, the corresponding median PFS (95% CI) was 4.17 months (2–NA), 2.57 months (2.37–NA), 4.2 months (3.23–5.37), and 2.47 months (2.03–3.5), respectively (Figure S8E). Moreover, the *KMT2C*/*TP53* co‐MUT subgroup tended to show an equal or higher rate of DCB relative to the *TP53*‐WT & *KMT2C*‐MUT, *TP53*‐MUT & *KMT2C*‐WT, and *KMT2C*/*TP53* co‐WT subgroups (40%, 40%, 32.1%, and 23.3%, respectively; *p* = 0.3) (Figure S8F).

It is well‐known that the *TP53*/*KRAS* co‐mutation could serve as a potential predictive marker in guiding anti‐PD‐1/PD‐L1 immunotherapy in NSCLC.^19^ We validated the predictive effect of *TP53*/*KRAS* co‐mutation in the Rizvi cohort and found that the PFS of patients with *TP53*/*KRAS* co‐mutation was superior to that of *TP53*‐MUT/*KRAS*‐WT, *TP53*‐WT/*KRAS*‐MUT, and *TP53*/*KRAS* co‐WT patients (median PFS: 5.77, 3.6, 2.33, and 2.6 months, respectively, *p* = 0.006, Figure S7B). Furthermore, we found that *TP53*/*KRAS* co‐MUT and *TP53*/*KMT2C* co‐MUT patients were two distinct subgroups with little overlap, both in our study and in the Rizvi cohort (Figure S7C). Therefore, we next validated whether *TP53* mutation combined with *KRAS* or *KMT2C* mutations was a better predictive factor. The median PFS (95% CI) was 7.17 months (4.27–NA), 2.37 months (1.93–4.37), 3.5 months (2.7–4.73), and 2.57 months (1.93–5.27) in the *KMT2C*/*KRAS*‐MUT & *TP53*‐MUT, *KMT2C*/*KRAS*‐MUT & *TP53*‐WT, *KMT2C*/*KRAS*‐WT & *TP53*‐MUT, and *KMT2C*/*KRAS*‐WT & *TP53*‐WT patients, respectively, (*KMT2C*/*KRAS*‐MUT & *TP53*‐MUT vs. *KMT2C*/*KRAS*‐WT & *TP53*‐WT, adjusted HR: 0.46, 95% CI: 0.26–0.81, *p* = 0.0073) (Figure 3E and Table 2). Furthermore, the *KMT2C*/*KRAS*‐MUT & *TP53*‐MUT subgroup showed a significantly higher DCB than the *KMT2C*/*KRAS*‐MUT & *TP53*‐WT, *KMT2C*/*KRAS*‐WT & *TP53*‐MUT, and *KMT2C*/*KRAS*‐WT & *TP53*‐WT subgroups (51.2%, 28.1%, 26.4%, and 20%, respectively; *p* = 0.0089) (Figure 3F).

**TABLE 2:** Univariate and multivariate analyses of clinical parameters and co‐mutation status of *TP53* with *KMT2C* or *KRAS* on progression‐free survival

	Unadjusted HR (95% CI)	*p* value	Adjusted HR (95% CI)	*p* value
Gender				
Female	Reference		Reference	
Male	1.09 (0.83–1.44)	0.5420	1.09 (0.81–1.47)	0.5495
Age	1.00 (0.99–1.01)	0.9270	0.99 (0.98–1.01)	0.3535
Smoking status				
Ever	Reference		Reference	
Never	1.45 (1.03–2.03)	0.0321	1.30 (0.86–1.96)	0.2069
Treatment type				
Combination				
Monotherapy	1.87 (1.21–2.87)	0.0045	1.96 (1.26–3.08)	0.0031
TMB group				
<10 muts/Mb	Reference		Reference	
>=10 muts/Mb	0.73 (0.54–0.99)	0.0454	0.93 (0.66–1.32)	0.6961
Histological type				
Non‐Squamous	Reference		Reference	
Squamous	1.02 (0.68–1.53)	0.9240	0.88 (0.57–1.35)	0.5494
Co‐mutation status				
KMT2C/KRAS‐WT & TP53‐WT	Reference		Reference	
KMT2C/KRAS‐MUT & TP53‐WT	1.03 (0.98–1.54)	0.9148	1.02 (0.64–1.63)	0.9219
KMT2C/KRAS‐WT & TP53‐MUT	0.85 (0.58–1.24)	0.3926	0.88 (0.57–1.36)	0.5655
KMT2C/KRAS‐MUT & TP53‐MUT	0.41 (0.25–0.67)	0.0004	0.46 (0.26–0.81)	0.0073

## 4 DISCUSSION

In this study, we systematically integrated comprehensive genomic profiling with the expression of PD‐L1 and TMB to explore the association between gene alterations and biomarkers for ICIs. Our results demonstrated that the status of genomic alterations of several oncogenic driver genes, such as *EGFR*, *ALK*, and *ROS1*, as well as several frequently mutated genes in NSCLC (e.g., *TP53*), correlated with the expression of PD‐L1 and TMB. In addition, our results indicated that co‐mutations of *TP53* and *KMT2C* associated with higher TMB and could function as potential predictive biomarkers for ICBs. Furthermore, our data demonstrated that co‐mutation of *TP53* with *KMT2C* or *KRAS* might identify a larger patient population that might benefit from ICBs.

At present, expression of PD‐L1 determined by IHC remains the only validated biomarker that has demonstrated strong correlation with ICI response. However, there is variability in marker staining among the five approved PD‐L1‐detecting antibodies (22C3, 28–8, SP142, SP263, and 73–10).^38^ It was also reported that the rate of positive PD‐L1 expression (≥ 50%) in Chinese patients with NSCLC might be different from those in patients of Western countries.^39^ In our study, the proportions of patients with PD‐L1 < 1%, 1–49%, and ≥50% were 76.7%, 14.8%, and 8.5%, respectively, similar to a report from a Chinese population study, where patients with PD‐L1 < 1% accounted for 79% and patients with PD‐L1 ≥ 50% accounted for only 7.6% of lung adenocarcinoma patients.^40^ In addition, previous reports of the relationship between PD‐L1 and TMB have been conflicting, with some studies reporting no correlation,^31^, ^41^ but several recent studies reporting a small but positive association between PD‐L1 and TMB in NSCLC and other cancer types.^42^, ^43^, ^44^ Yarchoan M et al. examined 9887 individual specimens and found that there was a small but positive association between PD‐L1 expression and TMB (Pearson's coefficient 0.084, *p* < 10–16).^42^ Rizvi et al. also reported that across 1023 NSCLC specimens examined by MSK‐IMPACT and PD‐L1 testing, there was a minor correlation between PD‐L1 and TMB (Spearman rho =0.195).^44^ In addition, Lamberti et al. suggested that across 421 NSCLC specimens with PD‐L1 TPS ≥90% (N = 133) or <1% (N = 288), examined using NGS, high PD‐L1 expression in NSCLC was associated with high TMB (*p* < 0.001).^45^ In our study, there was a minor but positive association between PD‐L1 expression and TMB (Kendall's coefficient 0.179, *p* < 0.001). In conclusion, consistent with previous studies, TMB and PD‐L1 expression are weakly but significantly correlated (*p* < 0.05); therefore, TMB‐H and PD‐L1‐High can be considered independent populations in NSCLC. There are several reasons that may explain the discrepancies between the study results: 1. Studies reporting no correlation between PD‐L1 and TMB included patients treated in clinical trials, which had limited sample sizes and representativeness, while studies reporting a small but positive association between PD‐L1 and TMB had larger sample sizes. 2. Different anti‐PD‐L1 monoclonal antibodies including 22C3, 28–8, E1L3 N, and others were used across these studies, which might contribute to the discrepancy. 3. Different TMB assessment methods were applied across these studies including whole‐exome sequencing (WES), MSK‐IMPACT, and FoundationOne, which also might contribute to the discrepancy.

Noted, *EGFR* is the most common oncogenic driver gene among Chinese patients with NSCLC and its mutation frequency in Chinese patients (~50%) is significantly higher than that in Western patients.^40^ Both *EGFR* mutations and *ALK* rearrangements have been suggested to be associated with lower expression of PD‐L1,^16^ lower TMB, and lower overall response rate to ICIs.^40^ Consistent with previous reports, our study data showed that *EGFR* actionable mutations were negatively associated with TMB, expression of PD‐L1 (Figure 1A and 1B), and clinical outcomes to ICI therapy (Figure S7D); *ALK* rearrangements were significantly correlated with lower TMB, but had no significant relationship with the expression of PD‐L1 and clinical ICI outcomes due to the small sample size (data not shown). Therefore, patients with *EGFR* mutations and *ALK* rearrangements were excluded in most immunotherapy trials. However, the results of the ATLANTIC study (n = 444) demonstrated that high PD‐L1‐expressing (≥ 25%) and *EGFR*/*ALK* mutation‐positive advanced NSCLC patients might have greater benefit from ICI treatment with durable efficacy and a promising effect on OS than patients with PD‐L1 expression <25% and *EGFR*/*ALK* mutation (median OS: 13.3 vs. 9.9 months).^46^ Recently, Mazieres J et al. reported that the anti‐tumor activity of ICIs in advanced NSCLC with oncogenic driver alteration was lower in the patients with an *EGFR*/*ALK* alteration than those with a *KRAS* alteration. The objective response rate to ICIs was 12% in patients with *EGFR* alterations and 0% in patients with *ALK* rearrangements. In addition, PFS was significantly different across *EGFR* mutational subtypes with a T790 M, exon 19, exon 21, and other mutational subtypes having PFS of 1.4, 1.8, 2.5, and 2.8 months, respectively (*p* < 0.001).^47^ Consistently, data from the Rizvi cohort showed that patients with uncommon *EGFR* mutations had worse PFS than patients with wild‐type and *EGFR*‐sensitizing mutations (Figure S7E). These findings suggested that some subtypes of driver genes are correlated with ICI treatment outcomes.

Besides driver genes, epigenetic alterations (involving DNA methylation and histone modifications) play pivotal roles in tumor initiation, progression, metastasis, and immune evasion in lung cancers, making them also viable targets for therapy.^48^ Aberrant promoter methylation of genes, such as *CDKN2A*, mutL homolog 1 (*MLH1*), mutS homolog 2 (*MSH2*), APC regulator of Wnt signaling pathway (*APC*), retinoic acid receptor beta (*RARB*), and O‐6‐methylguanine‐DNA methyltransferase (*MGMT*) has been described in lung cancer.^49^ Furthermore, different chromatin modifications can be used as prognostic markers. For example, globally elevated H3 and H4 methylation was shown to be associated with poor prognosis, whereas high dimethyl H3 K4 levels appeared to confer a better prognosis.^50^ Histone methylation marks are deposited by KMTs, which can be divided into non‐SET and SET domain‐containing KMTs (e.g., KMT2A‐D, SET1A/B, and SET7/9). For instance, KMT2C (also known as MLL3) belongs to the mixed‐lineage leukemia (MLL) family of histone methyltransferases, which mono‐methylate H3 K4 at enhancers as part of the complex proteins associated with the Set1 (COMPASS) complex.^51^ Accordingly, KMT2C has been reported as a tumor repressor frequently altered in several types of cancers, including myeloid leukemia, colorectal cancer, medulloblastoma, pancreatic ductal adenocarcinoma, glioblastoma, and lung adenocarcinoma.^28^ Furthermore, a recent study demonstrated that downregulation of *KMT2C* compromised the homologous recombination‐mediated double‐strand break DNA repair function in several cancer types, including NSCLC. This induced substantially higher genomic instability which was linked to higher TMB and sensitizing cancer cells to ICIs.^27^, ^52^ These findings suggested that aberrant genomic alterations of *KMT2C* could pose as a potential target for lung cancer therapy. In this study, we found that patients with *KMT2C* mutations exhibited a higher TMB and longer PFS with ICI treatments than did those with wild‐type *KMT2C* (Figure 2F and 2I). Additionally, we ran an in silico mutation prediction analyses using PolyPhen‐2 to predict the functional impact of *KMT2C* mutations and listed the functional information of *KMT2C* mutations from the OrigiMed and cBioportal data sets in Tables S4 and S5. Then, we reassessed the survival and genomic analysis with only potentially damaging *KMT2C* mutations and the results showed that the predicted loss‐of‐function *KMT2C* mutations were associated with higher TMB but not correlated with PD‐L1 expression; patients with potentially damaging *KMT2C* mutations had a trend for better PFS than those with *KMT2C*‐WT (median, 4.08 vs. 3.17 months, *p* = 0.25), which is consistent with the data in Figure 2 (Figure S8).

A previous report indicated that oncogenic driver genes can modulate tumor immune microenvironment, especially in NSCLC.^53^ Nevertheless, single gene alterations, such as *TP53*, might be positively associated with PD‐L1 expression and immune‐related genes expression, but single gene alteration alone was not able to distinguish responders from receiving ICI treatments in NSCLC; however, patients with *TP53*/*KRAS* co‐mutations were reported to be sensitive to ICI treatments, suggesting the necessity for implementation of a model combining multiple genes.^19^ Recently, a *STK11*/*LKB1* co‐mutation in *KRAS*‐mutant NSCLC was reported as a new predictive marker for tumor resistance to ICI therapy. A *KRAS*/*STK11* co‐alteration was correlated with significantly shorter PFS after ICI treatments compared with *KRAS* alteration alone (PFS HR, 1.98; *p* < 0.001) or a *KRAS*/*TP53* co‐alteration (PFS HR, 1.77; *p* = 0.0072).^54^ Another study reported that a *KRAS*/*KEAP1* co‐alteration is an independent prognostic factor, predicting inferior survival (HR, 1.96; 95% CI, 1.33–2.92; *p* < 0.001), duration of response to initial platinum‐based chemotherapy (HR, 1.64; 95% CI, 1.04–2.59; *p* = 0.03), and OS from the start of immunotherapy (HR, 3.54; 95% CI, 1.55–8.11; *p* = 0.003).^55^ In our study, KMT2C and *TP53* co‐mutation could serve as a better biomarker for predicting the PFS and DCB of ICI therapy than *KMT2C* mutations alone, whereas *KMT2A* or *KMT2D* with *TP53* co‐mutations might not distinguish patients potentially benefiting from ICI treatments (Figure S6I and S6J). Similarly, another study reported increased sensitivity to PD‐1 blockade in patients with *TP53* and *KRAS* mutations. However, not all patients with *TP53*‐ or *KRAS*‐mutated tumors or both responded to this treatment. Furthermore, our data showed that *KMT2C*‐MUT/*TP53*‐MUT and *KRAS*‐MUT/*TP53*‐MUT patients comprised two distinct populations with little overlap (Figure S7C). Therefore, *KRAS* was recruited into our biomarker combination and patients with co‐occurring mutations of *TP53* and *KMT2C* or *KRAS* showed remarkable clinical benefit from ICI treatments (Figure 3E‐F and Table 2).

It is a well‐accepted tendency that a combination of biomarkers for each process could provide complementary information affording greater accuracy in the prediction of immunotherapeutic benefit. Recent work has demonstrated that combination of TMB with GEP, a T‐cell–inflamed gene expression profile, can jointly predict clinical responses to pembrolizumab in pan‐tumor types and identify patterns of underlying, targetable biology related to these groups.^56^ Accordingly, we discovered that *KMT2C*/*TP53* co‐mutation and PD‐L1 could function as independent predictors (Figure 3B) demonstrating low correlation, and as expected, they might exhibit joint predictive power in stratifying responders from non‐responders. Since PD‐L1 protein expression assay has been developed for clinical use, apart from being rational, implementation of our model in clinical utility is feasible and also quite promising, as it only includes a small panel of two genes.

The main limitation of our study is that these findings originated from a retrospective profiling analysis and were not able to be validated in Chinese NSCLC ICI‐treated cohorts. Although our findings were validated by the Rizvi cohort derived from cBioPortal, the sample size of this cohort was relatively small (patients with *KMT2C* and *TP53* co‐mutation were even fewer) and it lacked information about important confounding factors such as performance status and presence of distant metastases in the viscera, brain, or bones. Based on this preliminary evidence, a future prospective, multicenter study for ICI response with larger sample sizes of *KMT2C*/*TP53* mutations, TMB, PD‐L1 expression, and TILs is warranted. Second, as ICI therapies together with chemotherapy are currently the first‐line standard treatment schemes for most patients, we regret that we do not have access to clinical outcome parameters of patients treated with chemo‐immuno‐therapies. As such, the predictive effect of *KMT2C*/*TP53* co‐mutation for chemo‐immuno‐therapies could not be validated in our study and we hope to investigate this in the future.

In conclusion, to the best of our knowledge, this is the largest analysis of Chinese NSCLC genomic profiling that integrates the expression of PD‐L1 and TMB. Our findings provide insight into the immune modulation controlled by certain driver genes, tumor suppressor genes, and epigenetic genes in NSCLC. Ultimately, our study demonstrated that *KMT2C*/*TP53* co‐mutation might be a potential biomarker to predict responses to PD‐1 blockade therapy in patients with NSCLC, and that adding *KRAS* to the biomarker combination might create a more robust parameter to identify the best responders to ICI therapy. Our study might increase knowledge toward further individualization of therapeutic decisions based on genetic biomarker information and is simple to implement in the real‐world setting. Ongoing intense work is attempting to further validate our model in large cohorts, and prospective clinical trials, especially in regard to chemo‐immunotherapy treatments.

## 5 COMPETING INTERESTS

The authors declare that they have no competing interests.

## ETHICS APPROVAL

This study was approved by the Institution Review Board of the First Hospital of Kunming Medical University and Guangdong Provincial People's Hospital, according to the Declaration of Helsinki. Informed consent was obtained from all enrolled patients.

## CONSENT FOR PUBLICATION

Not applicable.

## Supporting information

Fig S1‐S8

Table S1‐S5

## Data Availability

The datasets used and/or analyzed during the current study are available from the corresponding author on reasonable request.
