# Supplementary material for: Integration of comprehensive genomic profiling, tumor mutational burden, and PD‐L1 expression to identify novel biomarkers of immunotherapy in non‐small cell lung cancer
Source: Cancer Med. 2021 Mar 2;10(7):2216–31. doi: 10.1002/cam4.3649 (PMC7982619; doi:10.1002/cam4.3649)
Supplement: Supplementary file 1 — Fig S1‐S8 [file CAM4-10-2216-s001.pdf]

Figure S1

A

B

C

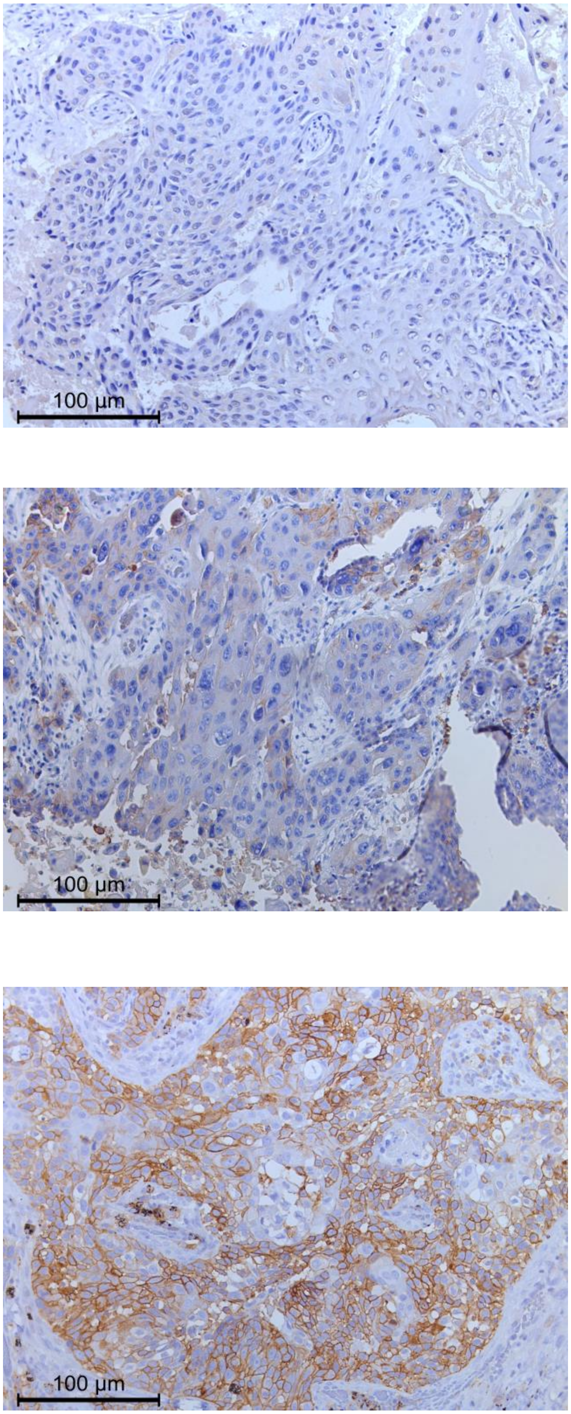

PD-L1 TPS
< 1%

PD-L1 TPS
1-49%

PD-L1 TPS
>= 50%

Figure S2

The Kendall Correlation Coefficient was 0.179 (p = 1.075e-08)

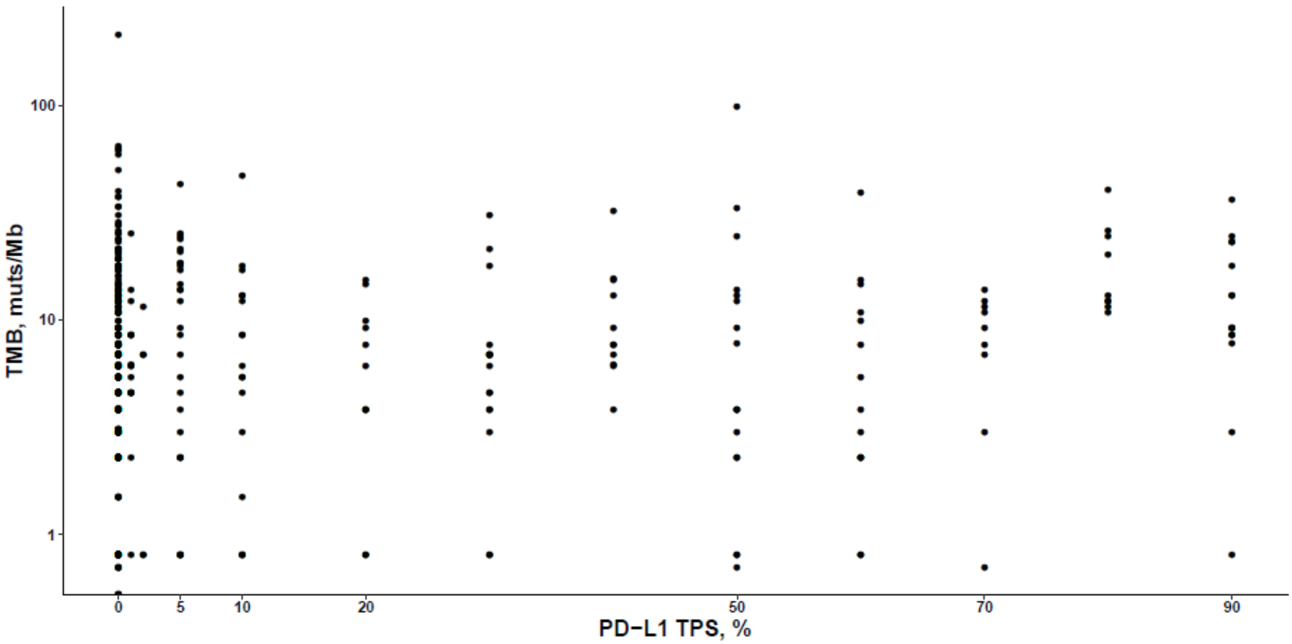

Figure S3

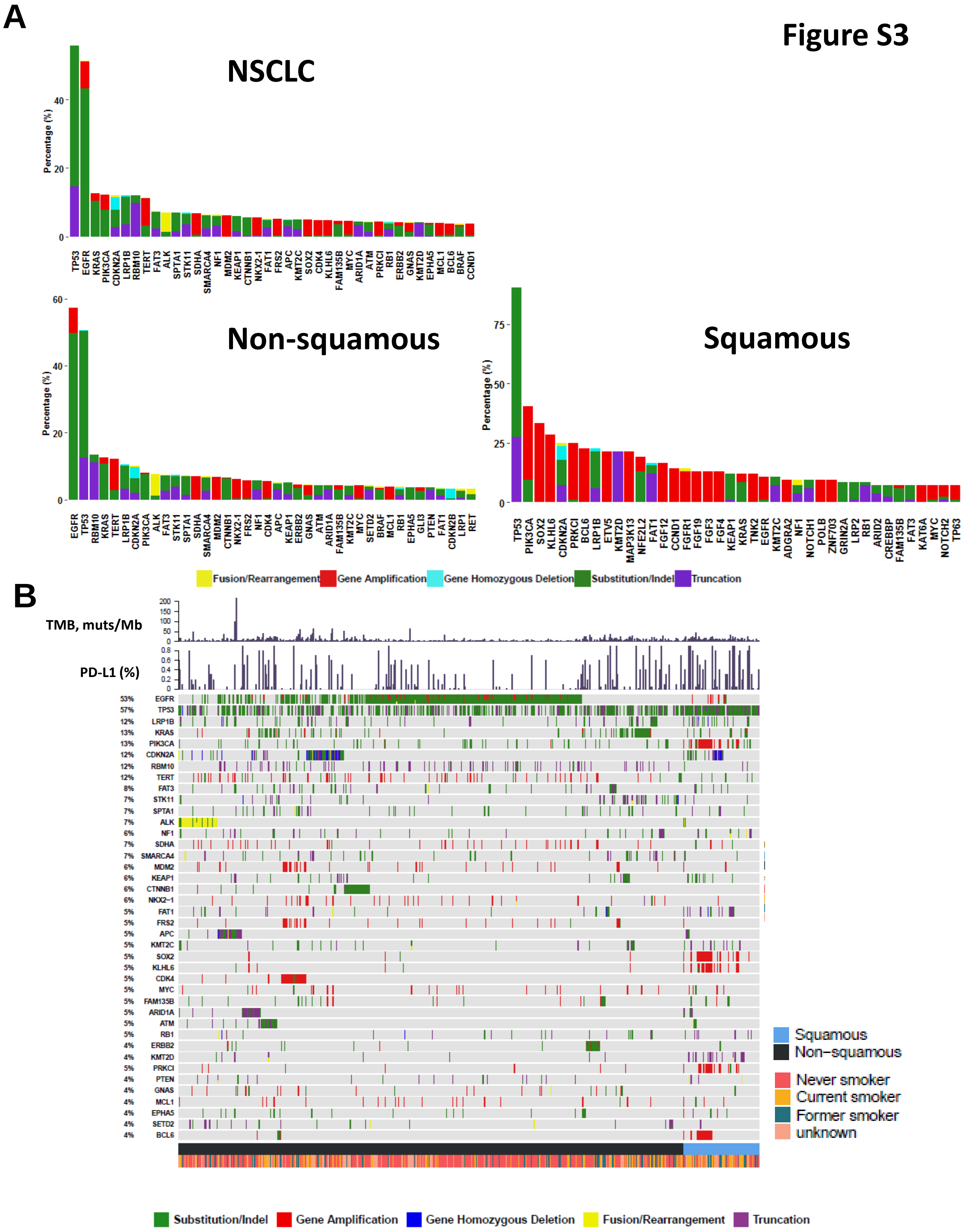
A
NSCLC
Non-squamous
Squamous
Percentage (%)
Fusion/Rearrangement
Gene Amplification
Gene Homozygous Deletion
Substitution/Indel
Truncation
B
TMB, muts/Mb
PD-L1 (%)
Squamous
Non−squamous
Never smoker
Current smoker
Former smoker
unknown
Substitution/Indel
Gene Amplification
Gene Homozygous Deletion
Fusion/Rearrangement
Truncation

Figure S4

A

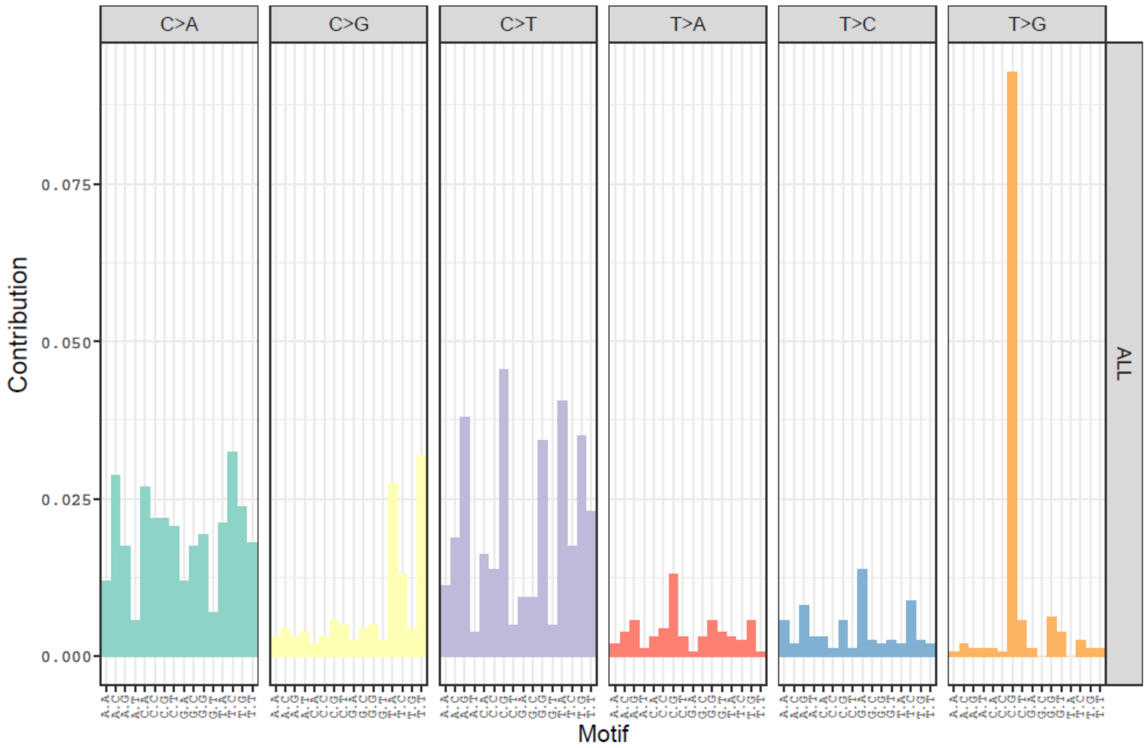
C>A
C>G
C>T
T>A
T>C
T>G
ALL
Contribution
0.075
0.050
0.025
0.000
Motif

B

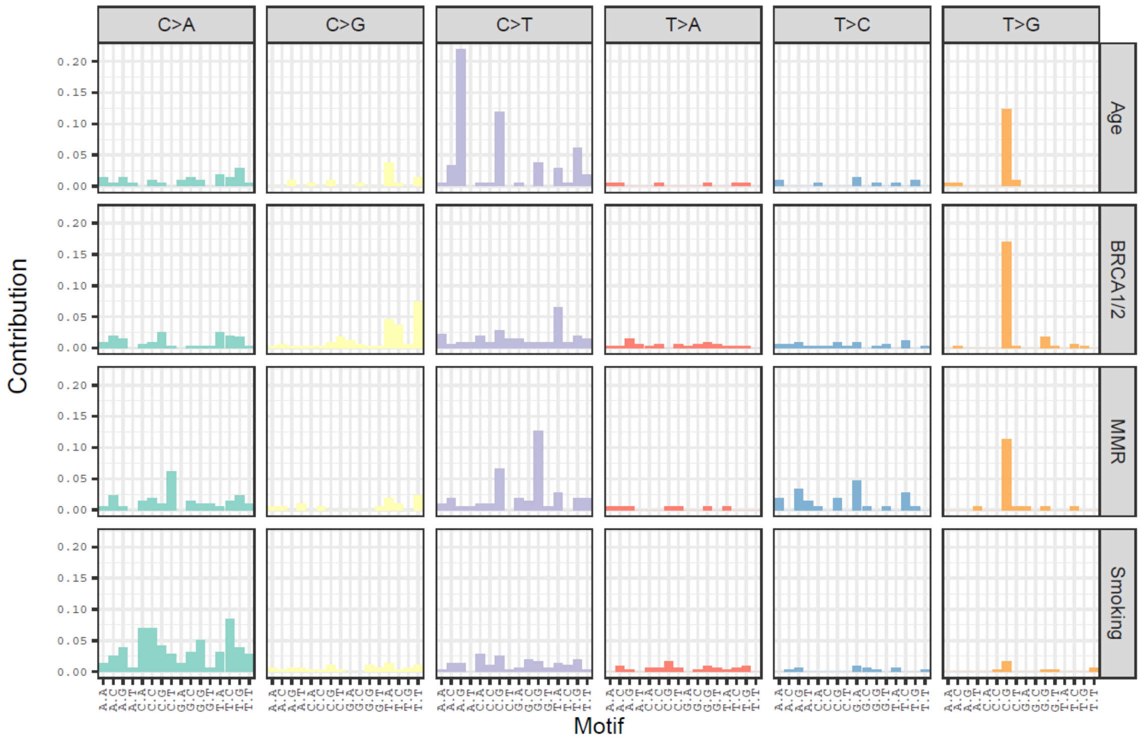
C>A
C>G
C>T
T>A
T>C
T>G
Age
BRCA1/2
MMR
Smoking
Contribution
0.20
0.15
0.10
0.05
0.00
Motif

Figure S5

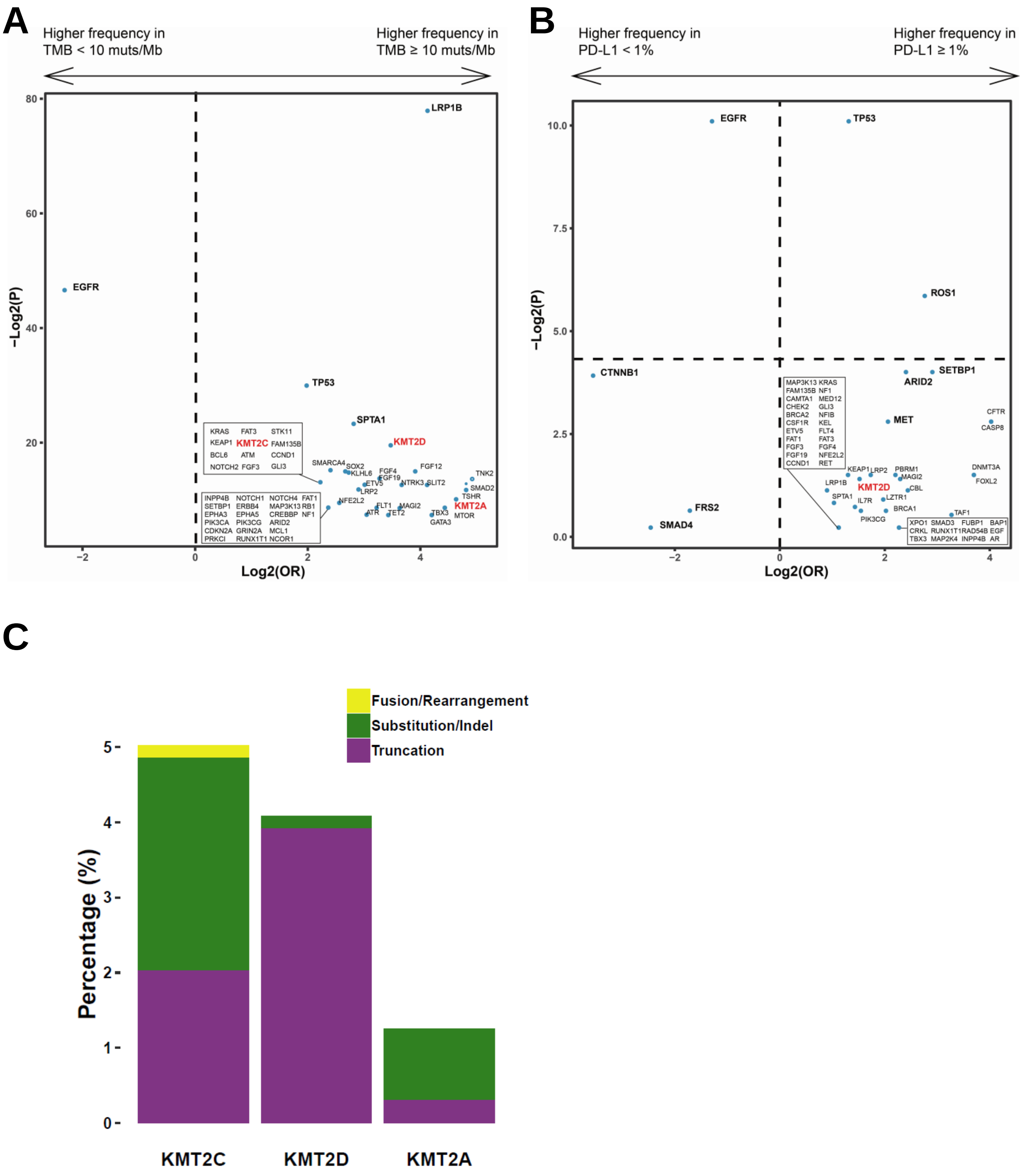
A
Higher frequency in TMB < 10 muts/Mb
Higher frequency in TMB ≥ 10 muts/Mb
−Log2(P)
Log2(OR)
LRP1B
EGFR
TP53
SPTA1
KMT2D
KMT2C
KMT2A
KRAS FAT3 STK11
KEAP1 KMT2C FAM135B
BCL6 ATM CCND1
NOTCH2 FGF3 GLI3
INPP4B NOTCH1 NOTCH4 FAT1
SETBP1 ERBB4 MAP3K13 RB1
EPHA3 EPHA5 CREBBP NF1
PIK3CA PIK3CG ARID2
CDKN2A GRIN2A MCL1
PRKCI RUNX1T1 NCOR1
SMARCA4
SOX2
KLHL6
FGF4
FGF19
FGF12
ETV5
LRP2
NTRK3
SLIT2
TNK2
SMAD2
TSHR
NFE2L2
FLT1
MAGI2
ATR
TET2
TBX3
GATA3
MTOR
B
Higher frequency in PD-L1 < 1%
Higher frequency in PD-L1 ≥ 1%
−Log2(P)
Log2(OR)
EGFR
TP53
ROS1
CTNNB1
SETBP1
ARID2
MET
CFTR
CASP8
MAP3K13 KRAS
FAM135B NF1
CAMTA1 MED12
CHEK2 GLI3
BRCA2 NFIB
CSF1R KEL
ETV5 FLT4
FAT1 FAT3
FGF3 FGF4
FGF19 NFE2L2
CCND1 RET
KEAP1
LRP2
PBRM1
MAGI2
LRP1B
KMT2D
CBL
DNMT3A
FOXL2
SPTA1
IL7R
LZTR1
PIK3CG
BRCA1
TAF1
FRS2
SMAD4
XPO1 SMAD3 FUBP1 BAP1
CRKL RUNX1T1 RAD54B EGF
TBX3 MAP2K4 INPP4B AR
C
Fusion/Rearrangement
Substitution/Indel
Truncation
Percentage (%)
KMT2C
KMT2D
KMT2A

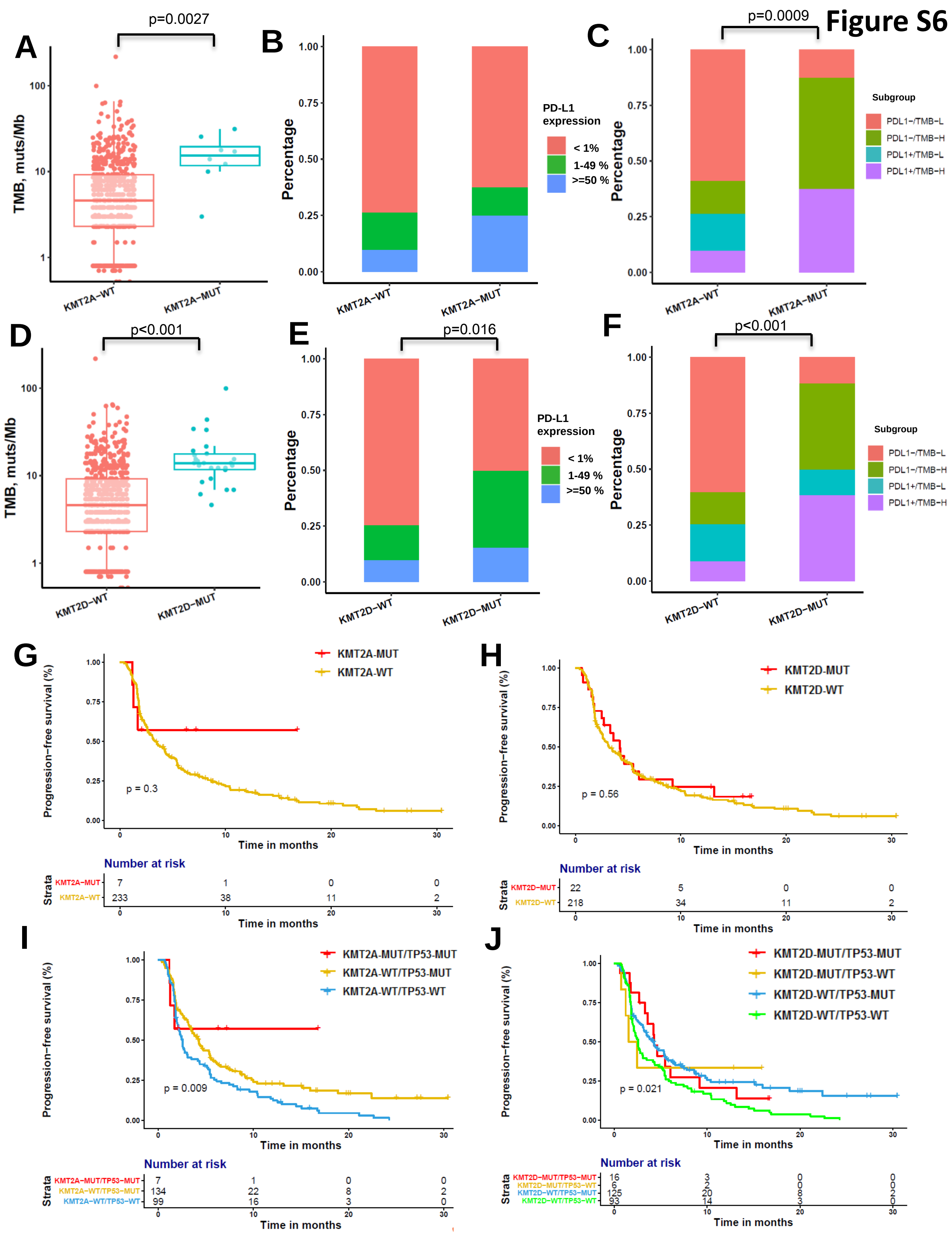

Figure S6
A
p=0.0027
TMB, muts/Mb
KMT2A−WT
KMT2A−MUT
B
Percentage
PD-L1 expression
< 1%
1-49 %
>=50 %
C
p=0.0009
Subgroup
PDL1−/TMB−L
PDL1−/TMB−H
PDL1+/TMB−L
PDL1+/TMB−H
D
p<0.001
KMT2D−WT
KMT2D−MUT
E
p=0.016
F
p<0.001
G
Progression−free survival (%)
KMT2A-MUT
KMT2A-WT
p = 0.3
Time in months
Number at risk
Strata
KMT2A−MUT 7 1 0 0
KMT2A−WT 233 38 11 2
H
KMT2D-MUT
KMT2D-WT
p = 0.56
KMT2D−MUT 22 5 0 0
KMT2D−WT 218 34 11 2
I
KMT2A-MUT/TP53-MUT
KMT2A-WT/TP53-MUT
KMT2A-WT/TP53-WT
p = 0.009
KMT2A−MUT/TP53−MUT 7 1 0 0
KMT2A−WT/TP53−MUT 134 22 8 2
KMT2A−WT/TP53−WT 99 16 3 0
J
KMT2D-MUT/TP53-MUT
KMT2D-MUT/TP53-WT
KMT2D-WT/TP53-MUT
KMT2D-WT/TP53-WT
p = 0.021
KMT2D−MUT/TP53−MUT 16 3 0 0
KMT2D−MUT/TP53−WT 6 2 0 0
KMT2D−WT/TP53−MUT 125 20 8 2
KMT2D−WT/TP53−WT 93 14 3 0

Figure S7

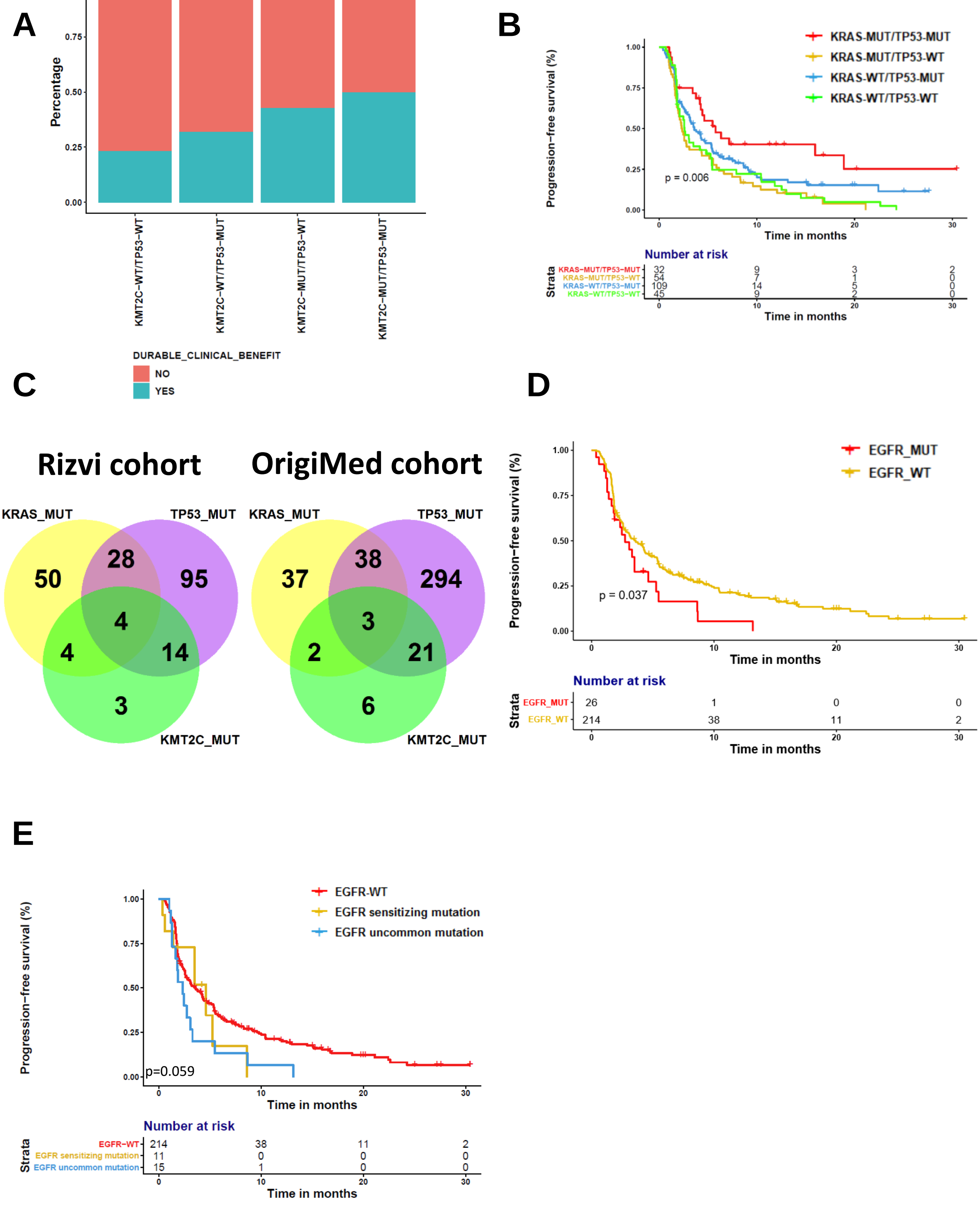
A
1.00
0.75
0.50
0.25
0.00
Percentage
KMT2C−WT/TP53−WT
KMT2C−WT/TP53−MUT
KMT2C−MUT/TP53−WT
KMT2C−MUT/TP53−MUT
DURABLE_CLINICAL_BENEFIT
NO
YES
B
KRAS-MUT/TP53-MUT
KRAS-MUT/TP53-WT
KRAS-WT/TP53-MUT
KRAS-WT/TP53-WT
Progression−free survival (%)
p = 0.006
Time in months
Number at risk
Strata
KRAS−MUT/TP53−MUT 32 9 3 2
KRAS−MUT/TP53−WT 54 7 1 0
KRAS−WT/TP53−MUT 109 14 5 0
KRAS−WT/TP53−WT 45 9 2 0
0 10 20 30
Time in months
C
Rizvi cohort
KRAS_MUT
TP53_MUT
KMT2C_MUT
50 28 95 4 4 14 3
OrigiMed cohort
KRAS_MUT
TP53_MUT
KMT2C_MUT
37 38 294 3 2 21 6
D
EGFR_MUT
EGFR_WT
Progression−free survival (%)
p = 0.037
Time in months
Number at risk
Strata
EGFR_MUT 26 1 0 0
EGFR_WT 214 38 11 2
0 10 20 30
Time in months
E
EGFR-WT
EGFR sensitizing mutation
EGFR uncommon mutation
Progression−free survival (%)
p=0.059
Time in months
Number at risk
Strata
EGFR−WT 214 38 11 2
EGFR sensitizing mutation 11 0 0 0
EGFR uncommon mutation 15 1 0 0
0 10 20 30
Time in months

Figure S8

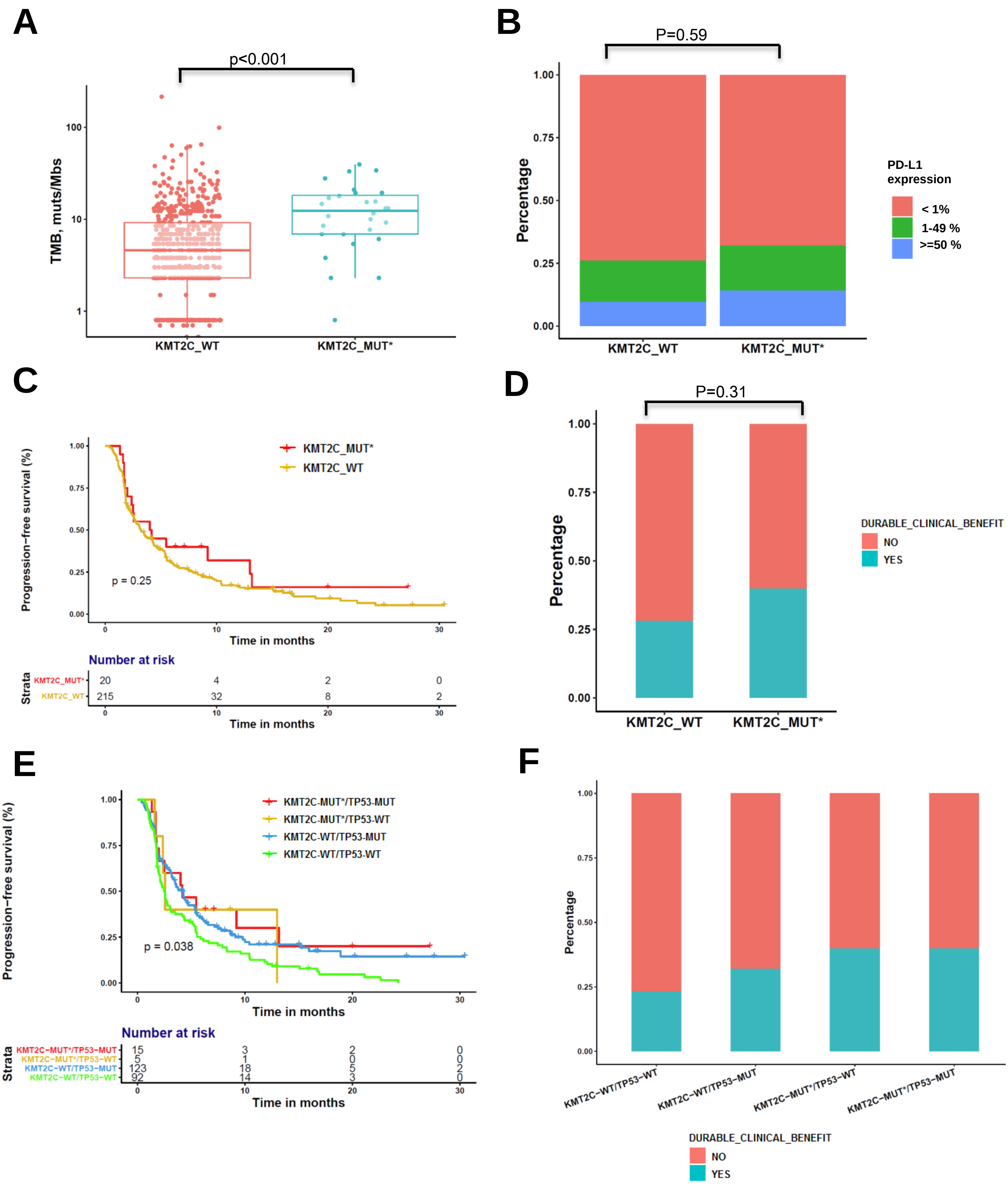

A
p<0.001
TMB, muts/Mbs
100
10
1
KMT2C_WT
KMT2C_MUT*
B
P=0.59
Percentage
1.00
0.75
0.50
0.25
0.00
KMT2C_WT
KMT2C_MUT*
PD-L1 expression
< 1%
1-49 %
>=50 %
C
Progression-free survival (%)
KMT2C_MUT*
KMT2C_WT
p = 0.25
Time in months
Number at risk
Strata
KMT2C_MUT* 20 4 2 0
KMT2C_WT 215 32 8 2
Time in months
D
P=0.31
Percentage
KMT2C_WT
KMT2C_MUT*
DURABLE_CLINICAL_BENEFIT
NO
YES
E
Progression-free survival (%)
KMT2C-MUT*/TP53-MUT
KMT2C-MUT*/TP53-WT
KMT2C-WT/TP53-MUT
KMT2C-WT/TP53-WT
p = 0.038
Time in months
Number at risk
Strata
KMT2C-MUT*/TP53-MUT 15 3 2 0
KMT2C-MUT*/TP53-WT 5 1 0 0
KMT2C-WT/TP53-MUT 123 18 5 2
KMT2C-WT/TP53-WT 92 14 3 0
Time in months
F
Percentage
KMT2C-WT/TP53-WT
KMT2C-WT/TP53-MUT
KMT2C-MUT*/TP53-WT
KMT2C-MUT*/TP53-MUT
DURABLE_CLINICAL_BENEFIT
NO
YES
