# Supplementary material for: Integration of comprehensive genomic profiling, tumor mutational burden, and PD‐L1 expression to identify novel biomarkers of immunotherapy in non‐small cell lung cancer
Source: Cancer Med. 2021 Mar 2;10(7):2216–31. doi: 10.1002/cam4.3649 (PMC7982619; doi:10.1002/cam4.3649)
Supplement: Supplementary file 2 — Table S1‐S5 [file CAM4-10-2216-s002.docx]

**Supplementary Table S1. Molecular characteristics of 637 NSCLC tumors and corresponding patient demographics**

|  | **Total, n (%)** | **PD-L1 TPS, %**  **(n=637)** | | | | **TMB, mut/Mb**  **(n=637)** | | |
| --- | --- | --- | --- | --- | --- | --- | --- | --- |
|  |  | < 1% | 1-49% | ≥50% | p value | Low (＜10) | High (≥10) | p value |
| **n (% of total)** | 637 | 468 (73.5) | 105 (16.5) | 64 (10) |  | 475 (70.6) | 162 (29.4) |  |
| **Age, years (median, IQR)** | 60 (53~67) | 60 (53~67) | 61 (49~68) | 62 (55~67) | 0.656 | 59 (53~66) | 62.5 (55~70) | <0.001 |
| **Age, years (mean±SD)** | 59.5±10.4 | 59.5±10.4 | 59.1±12.3 | 60.3±10.9 | 0.787 | 58.6±10.8 | 62.3±10.3 | <0.001 |
| **Gender, n (%)** |  |  |  |  | 0.002 |  |  | <0.001 |
| **Male** | 355 (55.7) | 243 (51.9) | 65 (61.9) | 47 (73.4) |  | 217 (45.7) | 138 (85.2) |  |
| **Female** | 282 (44.3) | 225 (48.1) | 40 (38.1) | 17 (26.6) |  | 258 (54.3) | 24 (14.8) |  |
| **Histology, n (%)** |  |  |  |  | <0.001 |  |  | <0.001 |
| **Squamous** | 84 (13.2) | 44 (9.4) | 23 (21.9) | 17 (26.6) |  | 39 (8.2) | 45 (27.8) |  |
| **Non-squamous** | 553 (86.8) | 424 (90.6) | 82 (78.1) | 47 (73.4) |  | 436 (91.8) | 117 (72.2) |  |
| **Smoking history, n (%)** |  |  |  |  | 0.2 |  |  | <0.001 |
| **Yes** | 203 (31.9) | 138 (29.5) | 38 (36.2) | 27 (42.1) |  | 112 (23.6) | 91 ( 56.2) |  |
| **No** | 345 (54.2) | 263 (56.2) | 54 (51.4) | 28 (43.8) |  | 304 (64.0) | 41 ( 25.3) |  |
| **Unknown** | 89 (13.9) | 67 (14.3) | 13 (12.4) | 9 (14.1) |  | 59 (12.4) | 30 ( 18.5) |  |
| **Stage at Dx, n (%)** |  |  |  |  | <0.001 |  |  | 0.038 |
| **Stage I** | 211 (33.1) | 184 (39.3) | 19 (18.1) | 8 (12.5) |  | 171 (36.0) | 40 ( 24.7) |  |
| **Stage II** | 64 (10) | 41 (8.8) | 14 (13.3) | 9 (14.1) |  | 43 ( 9.1) | 21 ( 13.0) |  |
| **Stage III** | 138 (21.7) | 87 (18.6) | 31 (29.5) | 20 (31.2) |  | 96 (20.2) | 42 ( 25.9) |  |
| **Stage IV** | 224 (35.2) | 156 (33.3) | 41 (39.1) | 27 (42.2) |  | 165 (34.7) | 59 ( 36.4) |  |
| **Distant metastasis, n (%)** |  |  |  |  | 0.106 |  |  | 0.697 |
| **Yes** | 217 (34) | 152 (32.5) | 40 (38.1) | 25 (39.1) |  | 160 (33.7) | 57 ( 35.2) |  |
| **No** | 415 (65) | 313 (66.9) | 65 (61.9) | 37 (57.8) |  | 312 (65.7) | 103 ( 63.6) |  |
| **Unknown** | 5 (1) | 3 (0.6) | 0 | 2 (3.1) |  | 3 ( 0.6) | 2 (1.2) |  |
| **TMB, mut/Mb (median, IQR)** | 4.6 (2.3~10) | 4.25 (2.3~8.5) | 6.9 (3.8~13.1) | 9.2 (3.6~13.9) | <0.001 | 3.8 (1.5~6.1) | 14.7 (12.3~21.6) | <0.001 |

**Supplementary Table S2. Correlation between mutation status of common driver genes and the combined biomarker of PD-L1 expression and TMB**

| **Genetic alteration** | **n (%)** |  |  | **Group (n=637)** |  | **p value** |
| --- | --- | --- | --- | --- | --- | --- |
|  |  | **PDL1-/TMB-L** | **PDL1-/TMB-H** | **PDL1+/TMB-L** | **PDL1+/TMB-H** |  |
| **n (% of total)** | 637 | 371 (58.2) | 97 (15.3) | 104 (16.3) | 65 (10.2) |  |
| **EGFR alteration status** |  |  |  |  |  | <0.001 |
| Wild type | 310 (48.7) | 127 (34.2) | 76 (78.4) | 59 (56.7) | 48 (73.8) |  |
| Mutated | 327 (51.3) | 244 (65.8) | 21 (21.6) | 45 (43.3) | 17 (26.2) |  |
| **ALK alteration status** |  |  |  |  |  | 0.621 |
| Wild type | 592 (92.9) | 344 (92.7) | 93 (95.9) | 95 (91.3) | 60 (92.3) |  |
| Mutated | 45 (7.1) | 27 (7.3) | 4 (4.1) | 9 (8.7) | 5 (7.7) |  |
| **BRAF alteration status** |  |  |  |  |  | 0.618 |
| Wild type | 613 (96.2) | 357 (96.2) | 92 (94.8) | 102 (98.1) | 62 (95.4) |  |
| Mutated | 24 (3.8) | 14 (3.8) | 5 (5.2) | 2 (1.9) | 3 (4.6) |  |
| **ERBB2 alteration status** |  |  |  |  |  | 0.829 |
| Wild type | 611 (95.9) | 354 (95.4) | 93 (95.9) | 100 (96.2) | 64 (98.5) |  |
| Mutated | 26 (4.1) | 17 (4.6) | 4 (4.1) | 4 (3.8) | 1 (1.5) |  |
| **MET alteration status** |  |  |  |  |  | 0.001 |
| Wild type | 619 (97.2) | 367 (98.9) | 95 (97.9) | 98 (94.2) | 59 (90.8) |  |
| Mutated | 18 (2.8) | 4 (1.1) | 2 (2.1) | 6 (5.8) | 6 (9.2) |  |
| **RET alteration status** |  |  |  |  |  | 0.007 |
| Wild type | 618 (97) | 362 (97.6) | 95 (97.9) | 103 (99) | 58 (89.2) |  |
| Mutated | 19 (3) | 9 (2.4) | 2 (2.1) | 1 (1) | 7 (10.8) |  |
| **ROS1 alteration status** |  |  |  |  |  | <0.001 |
| Wild type | 619 (97.2) | 367 (98.9) | 95 (97.9) | 94 (90.4) | 63 (96.9) |  |
| Mutated | 18 (2.8) | 4 (1.1) | 2 (2.1) | 10 (9.6) | 2 (3.1) |  |
| **NTRK alteration status** |  |  |  |  |  | 0.001 |
| Wild type | 615 (96.5) | 362 (97.6) | 90 (92.8) | 104 (100) | 59 (90.8) |  |
| Mutated | 22 (3.5) | 9 (2.4) | 7 (7.2) | 0 | 6 (9.2) |  |
| **NTRK1 alteration status** |  |  |  |  |  | 0.807 |
| Wild type | 632 (99.2) | 367 (98.9) | 96 (99) | 104 (100) | 65 (100) |  |
| Mutated | 5 (0.8) | 4 (1.1) | 1 (1) | 0 | 0 |  |
| **NTRK2 alteration status** |  |  |  |  |  | 0.637 |
| Wild type | 634 (99.5) | 369 (99.5) | 96 (99) | 104 (100) | 65 (100) |  |
| Mutated | 3 (0.5) | 2 (0.5) | 1 (1) | 0 | 0 |  |
| **NTRK3 alteration status** |  |  |  |  |  | <0.001 |
| Wild type | 622 (97.6) | 368 (97.6) | 91 (92.8) | 104 (100) | 59 (90.8) |  |
| Mutated | 15 (2.4) | 3 (2.4) | 6 (7.2) | 0 | 6 (9.2) |  |

**Supplementary Table S3. Correlation between mutation status of selected frequently mutated genes and the combined biomarker of PD-L1 expression and TMB**

| **Genetic alteration** | **n (%)** | **Group (n=637)** | | | | **p value** |
| --- | --- | --- | --- | --- | --- | --- |
|  |  | **PDL1-/TMB-L** | **PDL1-/TMB-H** | **PDL1+/TMB-L** | **PDL1+/TMB-H** |  |
| **n (% of total)** | 637 | 371 (58.2) | 97 (15.3) | 104 (16.3) | 65 (10.2) |  |
| **TP53 alteration status** |  |  |  |  |  | <0.001 |
| Wild type | 281 (44.1) | 210 (56.6) | 25 (25.8) | 36 (34.6) | 10 (15.4) |  |
| Mutated | 356 (55.9) | 161 (43.4) | 72 (74.2) | 68 (65.4) | 55 (84.6) |  |
| **KRAS alteration status** |  |  |  |  |  | <0.001 |
| Wild type | 557 (87.4) | 337 (90.8) | 77 (79.4) | 95 (91.3) | 48 (73.8) |  |
| Mutated | 80 (12.6) | 34 (9.2) | 20 (20.6) | 9 (8.7) | 17 (26.2) |  |
| **LRP1B alteration status** |  |  |  |  |  | <0.001 |
| Wild type | 560 (87.9) | 359 (96.8) | 61 (62.9) | 100 (96.2) | 40 (61.5) |  |
| Mutated | 77 (12.1) | 12 (3.2) | 36 (37.1) | 4 (3.8) | 25 (38.5) |  |
| **FAT1 alteration status** |  |  |  |  |  | 0.008 |
| Wild type | 604 (94.8) | 360 (97.0) | 87 (89.7) | 98 (94.2) | 59 (90.8) |  |
| Mutated | 33 (5.2) | 11 (3.0) | 10 (10.3) | 6 (5.8) | 6 (9.2) |  |
| **FAT3 alteration status** |  |  |  |  |  | <0.001 |
| Wild type | 590 (92.6) | 358 (96.5) | 82 (84.5) | 95 (91.3) | 55 (84.6) |  |
| Mutated | 47 (7.4) | 13 (3.5) | 15 (15.5) | 9 (8.7) | 10 (15.4) |  |
| **KMT2C alteration status** |  |  |  |  |  | <0.001 |
| Wild type | 605 (95) | 363 (97.8) | 84 (86.6) | 100 (96.2) | 58 (89.2) |  |
| Mutated | 32 (5) | 8 (2.2) | 13 (13.4) | 4 (3.8) | 7 (10.8) |  |
| **KMT2D alteration status** |  |  |  |  |  | <0.001 |
| Wild type | 611 (95.9) | 368 (99.2) | 87 (89.7) | 101 (97.1) | 55 (84.6) |  |
| Mutated | 26 (4.1) | 3 (0.8) | 10 (10.3) | 3 (2.9) | 10 (15.4) |  |
| **ARID1A alteration status** |  |  |  |  |  | 0.018 |
| Wild type | 609 (95.6) | 361 (97.3) | 89 (91.8) | 100 (96.2) | 59 (90.8) |  |
| Mutated | 28 (4.4) | 10 (2.7) | 8 (8.2) | 4 (3.8) | 6 (9.2) |  |
| **PIK3CA alteration status** |  |  |  |  |  | 0.005 |
| Wild type | 613 (96.2) | 335 (90.3) | 77 (79.4) | 95 (91.3) | 52 (80.0) |  |
| Mutated | 24 (3.8) | 36 (9.7) | 20 (20.6) | 9 (8.7) | 13 (20.0) |  |
| **KEAP1 alteration status** |  |  |  |  |  | <0.001 |
| Wild type | 599 (94) | 359 (96.8) | 88 (90.7) | 100 (96.2) | 52 (80.0) |  |
| Mutated | 38 (6) | 12 (3.2) | 9 (9.3) | 4 (3.8) | 13 (20.0) |  |
| **STK11 alteration status** |  |  |  |  |  | <0.001 |
| Wild type | 593 (93.1) | 357 (96.2) | 77 (79.4) | 98 (94.2) | 61 (93.8) |  |
| Mutated | 44 (6.9) | 14 (3.8) | 20 (20.6) | 6 (5.8) | 4 (6.2) |  |
| **CDKN2A alteration status** |  |  |  |  |  | 0.015 |
| Wild type | 560 (87.9) | 337 (90.8) | 78 (80.4) | 92 (88.5) | 53 (81.5) |  |
| Mutated | 77 (12.1) | 34 (9.2) | 19 (19.6) | 12 (11.5) | 12 (18.5) |  |

**Supplementary Table S4. The list of KMT2C mutations found in NSCLC and in silico mutation prediction analyses using PoliPhen-2 in the Origimed data set.**

| ORDER_ID | GENE | | Protein Change | DNA Change | Annotation | Mutation Type | Functional Impact |
| --- | --- | --- | --- | --- | --- | --- | --- |
| 118K6628M1 | KMT2C | | p.A4065V | c.12194C>T |  | Substitution/Indel | Polyphen-2: BENIGN, score: 0.226 |
| 118D6963A1 | KMT2C | p.E495D | | c.1485G>T |  | Substitution/Indel | Polyphen-2: BENIGN, score: 0.058 |
| 118G9831A1 | KMT2C | p.K834* | | c.2500A>T |  | Truncation | Polyphen-2: NA |
| 118J4270M1 | KMT2C | p.E2698G | | c.8093A>G |  | Substitution/Indel | Polyphen-2: POSSIBLY DAMAGING, score: 0.682 |
| 118G9407A1 | KMT2C | p.R2609* | | c.7825C>T |  | Truncation | Polyphen-2: NA |
| 118H1806D1 | KMT2C | p.R3177H | | c.9530G>A |  | Substitution/Indel | Polyphen-2: PROBABLY DAMAGING, score: 0.999 |
| 118H1806D1 | KMT2C | p.L804F | | c.2410C>T |  | Substitution/Indel | Polyphen-2: BENIGN, score: 0.002 |
| 118L7754D2 | KMT2C | p.E1734* | | c.5200G>T |  | Truncation | Polyphen-2: NA |
| 118G9334D1 | KMT2C | p.D1098Y | | c.3292G>T |  | Substitution/Indel | Polyphen-2: PROBABLY DAMAGING, score: 0.976 |
| 118H2324D1 | KMT2C | p.H365Q | | c.1095C>A |  | Substitution/Indel | Polyphen-2: PROBABLY DAMAGING, score: 0.998 |
| 118L7950D1 | KMT2C | p.C370* | | c.1110C>A |  | Truncation | Polyphen-2: NA |
| 118L7291M1 | KMT2C | p.T360S | | c.1079C>G |  | Substitution/Indel | Polyphen-2: PROBABLY DAMAGING, score: 0.997 |
| 118K5725M2 | KMT2C | p.G824C | | c.2470G>T |  | Substitution/Indel | Polyphen-2: PROBABLY DAMAGING, score: 0.999 |
| 117L3937A1 | KMT2C | p.L2451* | | c.7352_7354delinsAA |  | Truncation | Polyphen-2: NA |
| 118C5450D1 | KMT2C | p.G964D | | c.2891G>A |  | Substitution/Indel | Polyphen-2: PROBABLY DAMAGING, score: 0.999 |
| 118H2191A1 | KMT2C | p.Q979* | | c.2935C>T |  | Truncation | Polyphen-2: NA |
| 118H2403M1 | KMT2C | p.H3626Ifs*4 | | c.10876del |  | Truncation | Polyphen-2: NA |
| 118I2821A1 | KMT2C | p.W1671* | | c.5012G>A |  | Truncation | Polyphen-2: NA |
| 118I4022D2 | KMT2C | p.R3850L | | c.11549G>T |  | Substitution/Indel | Polyphen-2: PROBABLY DAMAGING, score: 0.999 |
| 118L7507M1 | KMT2C | - | | c.11813-2A>T | splicing variant | Substitution/Indel | Polyphen-2: NA |
| 118L7904D1 | KMT2C | p.Y138* | | c.414C>A |  | Truncation | Polyphen-2: NA |
| 118E7003D1 | KMT2C | p.T2819Ifs*5 | | c.8454_8455dup |  | Truncation | Polyphen-2: NA |
| 118I3008D1 | KMT2C | p.S3762C | | c.11285C>G |  | Substitution/Indel | Polyphen-2: POSSIBLY DAMAGING, score: 0.641 |
| 118K6327D1 | KMT2C | p.P837R | | c.2510C>G |  | Substitution/Indel | Polyphen-2: PROBABLY DAMAGING, score: 0.999 |
| 118F8242D1 | KMT2C | - | | DPP6-KMT2C | DPP6-KMT2C：rearrangement | Fusion/Rearrangement | Polyphen-2: NA |
| 118F8242D1 | KMT2C | - | | c.4379-1G>A | splicing variant | Substitution/Indel | Polyphen-2: NA |
| 118J5089D1 | KMT2C | p.Q1392K | | c.4174C>A |  | Substitution/Indel | Polyphen-2: BENIGN, score: 0.004 |
| 118J4725A1 | KMT2C | p.R4806* | | c.14416C>T |  | Truncation | Polyphen-2: NA |
| 119A9840J1 | KMT2C | p.K1665Gfs*5 | | c.4993_4994del |  | Truncation | Polyphen-2: NA |
| 118F8750D1 | KMT2C | p.W1639* | | c.4916G>A |  | Truncation | Polyphen-2: NA |
| 118K6496D1 | KMT2C | p.R1730C | | c.5188C>T |  | Substitution/Indel | Polyphen-2: PROBABLY DAMAGING, score: 0.999 |
| 118C5375D1 | KMT2C | - | | c.7443-205_7567del | long InDel（330bp deletion） | Substitution/Indel | Polyphen-2: NA |
| 118I3814A1 | KMT2C | p.P787L | | c.2360C>T |  | Substitution/Indel | Polyphen-2: BENIGN, score: 0.001 |
| 118C5291D1 | KMT2C | p.G363V | | c.1088G>T |  | Substitution/Indel | Polyphen-2: PROBABLY DAMAGING, score: 0.999 |

**Supplementary Table S5. The list of KMT2C mutations found in NSCLC and in silico mutation prediction analyses using PoliPhen-2 in the cBioportal data set.**

| Sample ID | Cancer Type | Protein Change | HGVSg | Functional Impact | Mutation Type |
| --- | --- | --- | --- | --- | --- |
| P-0012931-T01-IM5 | Lung Squamous Cell Carcinoma | E1333* | 7:g.151900114C>A | Polyphen-2: NA | Nonsense_Mutation |
| P-0009319-T01-IM5 | Lung Adenocarcinoma | R4690* | 7:g.151842344G>A | Polyphen-2: NA | Nonsense_Mutation |
| P-0003869-T01-IM5 | Lung Adenocarcinoma | W4357* | 7:g.151845941C>T | Polyphen-2: NA | Nonsense_Mutation |
| P-0001171-T01-IM3 | Lung Adenocarcinoma | X3125_splice | 7:g.151871215C>G | Polyphen-2: NA | Splice_Site |
| P-0004701-T01-IM5 | Non-Small Cell Lung Cancer | I4307Sfs*19 | 7:g.151846094del | Polyphen-2: NA | Frame_Shift_Del |
| P-0009833-T01-IM5 | Lung Adenocarcinoma | MLL3-intragenic | | Polyphen-2: NA | Fusion |
| P-0001171-T01-IM3 | Lung Adenocarcinoma | G892E | 7:g.151932996C>T | Polyphen-2: impact: probably_damaging, score: 0.999 | Missense_Mutation |
| P-0007101-T02-IM5 | Lung Adenocarcinoma | F2349L | 7:g.151877898G>C | Polyphen-2: impact: probably_damaging, score: 0.97 | Missense_Mutation |
| P-0006343-T01-IM5 | Lung Adenocarcinoma | E3845K | 7:g.151856085C>T | Polyphen-2: impact: probably_damaging, score: 0.985 | Missense_Mutation |
| P-0003229-T01-IM5 | Lung Adenocarcinoma | M3299I | 7:g.151860765C>T | Polyphen-2: impact: benign, score: 0.001 | Missense_Mutation |
| P-0007978-T01-IM5 | Lung Adenocarcinoma | R4864T | 7:g.151835933C>G | Polyphen-2: impact: probably_damaging, score: 0.936 | Missense_Mutation |
| P-0004472-T01-IM5 | Lung Adenocarcinoma | R2596L | 7:g.151874751C>A | Polyphen-2: impact: possibly_damaging, score: 0.932 | Missense_Mutation |
| P-0005848-T01-IM5 | Lung Adenocarcinoma | H493Q | 7:g.151949166G>T | Polyphen-2: impact: probably_damaging, score: 0.916 | Missense_Mutation |
| P-0002917-T01-IM3 | Lung Adenocarcinoma | L4699P | 7:g.151842316A>G | Polyphen-2: impact: probably_damaging, score: 0.986 | Missense_Mutation |
| P-0000082-T01-IM3 | Lung Adenocarcinoma | E2231G | 7:g.151878253T>C | Polyphen-2: impact: benign, score: 0.003 | Missense_Mutation |
| P-0001093-T01-IM3 | Lung Adenocarcinoma | K3714E | 7:g.151859522T>C | Polyphen-2: impact: benign, score: 0.015 | Missense_Mutation |
| P-0003562-T02-IM5 | Lung Adenocarcinoma | E3409D | 7:g.151860435C>G | Polyphen-2: impact: probably_damaging, score: 0.984 | Missense_Mutation |
| P-0009764-T01-IM5 | Lung Adenocarcinoma | A3211G | 7:g.151864349G>C | Polyphen-2: impact: probably_damaging, score: 0.986 | Missense_Mutation |
| P-0006954-T01-IM5 | Lung Adenocarcinoma | I1999M | 7:g.151878948T>C | Polyphen-2: impact: benign, score: 0.001 | Missense_Mutation |
| P-0001171-T01-IM3 | Lung Adenocarcinoma | D3386H | 7:g.151860506C>G | Polyphen-2: impact: probably_damaging, score: 0.91 | Missense_Mutation |
| P-0012427-T01-IM5 | Lung Adenocarcinoma | P2566S | 7:g.151874842G>A | Polyphen-2: impact: possibly_damaging, score: 0.555 | Missense_Mutation |
| P-0008848-T01-IM5 | Large Cell Neuroendocrine Carcinoma | E1356Q | 7:g.151900045C>G | Polyphen-2: impact: probably_damaging, score: 0.986 | Missense_Mutation |
| P-0012373-T01-IM5 | Lung Adenocarcinoma | R2235M | 7:g.151878241C>A | Polyphen-2: impact: possibly_damaging, score: 0.862 | Missense_Mutation |
| P-0015785-T01-IM6 | Lung Adenocarcinoma | S1414L | 7:g.151896396G>A | Polyphen-2: impact: possibly_damaging, score: 0.709 | Missense_Mutation |
| P-0012817-T01-IM5 | Lung Adenocarcinoma | G2280R | 7:g.151878107C>G | Polyphen-2: impact: benign, score: 0.086 | Missense_Mutation |
| P-0015531-T01-IM6 | Lung Squamous Cell Carcinoma | S3339C | 7:g.151860647T>A | Polyphen-2: impact: probably_damaging, score: 0.963 | Missense_Mutation |
| P-0015078-T01-IM6 | Lung Adenocarcinoma | R1934M | 7:g.151879144C>A | Polyphen-2: impact: probably_damaging, score: 0.996 | Missense_Mutation |
| P-0000012-T03-IM3 | Lung Adenocarcinoma | K3105N | 7:g.151871275T>A | Polyphen-2: impact: probably_damaging, score: 0.995 | Missense_Mutation |
